# Recent Advances in Surface Nanoengineering for Biofilm Prevention and Control. Part II: Active, Combined Active and Passive, and Smart Bacteria-Responsive Antibiofilm Nanocoatings

**DOI:** 10.3390/nano10081527

**Published:** 2020-08-04

**Authors:** Paul Cătălin Balaure, Alexandru Mihai Grumezescu

**Affiliations:** 1 “Costin Nenitzescu” Department of Organic Chemistry, Faculty of Applied Chemistry and Materials Science, University Politehnica of Bucharest, G. Polizu Street 1–7, 011061 Bucharest, Romania; paul.balaure@upb.ro; 2 Department of Science and Engineering of Oxide Materials and Nanomaterials, Faculty of Applied Chemistry and Materials Science, University Politehnica of Bucharest, G. Polizu Street 1–7, 011061 Bucharest, Romania

**Keywords:** biofilm-related pathogenic infections, contact-killing nanocoatings, drug-eluting nanocoatings, bacteria-triggered antimicrobial-releasing systems, inhibitors of bacterial signaling processes, dispersion of the biofilm matrix, combined active and passive strategies, antibiofilm platforms with reversibly switchable functionality between the non-fouling status and the bactericidal status, smart antibiofilm coatings, self-defensive coatings

## Abstract

The second part of our review describing new achievements in the field of biofilm prevention and control, begins with a discussion of the active antibiofilm nanocoatings. We present the antibiofilm strategies based on antimicrobial agents that kill pathogens, inhibit their growth, or disrupt the molecular mechanisms of biofilm-associated increase in resistance and tolerance. These agents of various chemical structures act through a plethora of mechanisms targeting vital bacterial metabolic pathways or cellular structures like cell walls and cell membranes or interfering with the processes that underlie different stages of the biofilm life cycle. We illustrate the latter action mechanisms through inhibitors of the quorum sensing signaling pathway, inhibitors of cyclic-di-GMP signaling system, inhibitors of (p)ppGpp regulated stringent response, and disruptors of the biofilm extracellular polymeric substances matrix (EPS). Both main types of active antibiofilm surfaces, namely non-leaching or contact killing systems, which rely on the covalent immobilization of the antimicrobial agent on the surface of the coatings and drug-releasing systems in which the antimicrobial agent is physically entrapped in the bulk of the coatings, are presented, highlighting the advantages of each coating type in terms of antibacterial efficacy, biocompatibility, selective toxicity, as well as drawbacks and limitations. Developments regarding combined strategies that join in a unique platform, both passive and active elements are not omitted. In such platforms with dual functionality, passive and active strategies can be applied either simultaneously or sequentially. We especially emphasize those systems that can be reversely and repeatedly switched between the non-fouling status and the bacterial killing status, thereby allowing several bacteria-killing/surface regeneration cycles to be performed without significant loss of the initial bactericidal activity. Eventually, smart antibiofilm coatings that release their antimicrobial payload on demand, being activated by various triggers such as changes in local pH, temperature, or enzymatic triggers, are presented. Special emphasis is given to the most recent trend in the field of anti-infective surfaces, specifically smart self-defensive surfaces for which activation and switch to the bactericidal status are triggered by the pathogens themselves.

## 1. Introduction

Passive antibiofilm strategies, which we presented in the first part of this review [1], target the attachment stage of the biofilm life cycle. They aim to prevent surface colonization by suppressing and/or weakening the intermolecular non-covalent forces developed during the primary non-specific reversible adhesion of biofoulants to biotic or abiotic surfaces.

The active antibiofilm strategies addressed herein use agents that either kill/inhibit bacterial growth or interfere with those key molecular mechanisms and signaling pathways, which underlie the processes of biofilm growth, maturation, and dispersal, and account for the remarkably increased antimicrobial resistance and tolerance of biofilm-associated pathogens. Active antibiofilm nanocoatings are presented and discussed in direct correlation with their chemical structures and mechanisms of action. Both currently used approaches, namely contact killing and drug-eluting, are illustrated with the most representative examples.

Since both passive (fouling resistant and fouling release [1]) and active (contact killing and drug-eluting) coatings have some drawbacks, scientists tried to enhance the antibiofilm performances by combining best aspects of each strategy in a single nanoplatform. Association of the two strategies will hopefully provide synergic benefits. There are two ways in which these unique nanocoatings integrating both passive antifouling and active action mechanisms can operate. The two strategies can be applied simultaneously or sequentially, and herein we provide illustrative examples of both variants. Furthermore, through adequate surface nanoengineering, these nanocoatings can be made to reversibly switch from the active contact-killing state to the passive non-fouling state and back several times.

Eventually, we address the newest trend in the field by presenting the attempts to fabricate smart active nanocoatings capable to sense small microenvironmental changes and to respond to these changings by releasing their therapeutic payload. This on-demand release can be triggered by pH, temperature, redox potential, or enzyme activity modifications. 

## 2. Antibiofilm Coatings Based on Polycationic Biocides

### 2.1. Tiggered Release Coatings

**Scheme 1:**
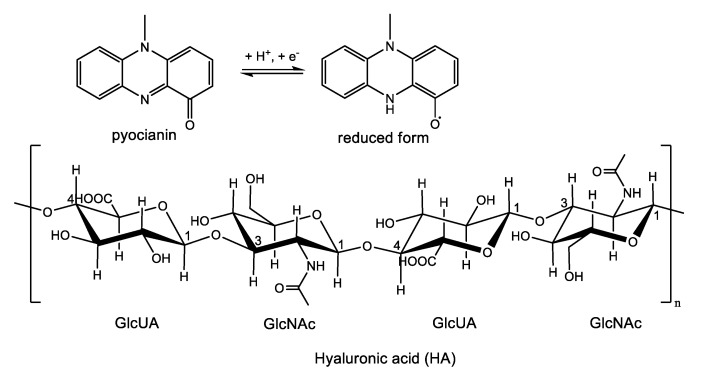
Structures of the pyocyanin and its reduced form and of the anionic polyelectrolyte hyaluronic acid (HA).

Cationic compounds are known as strong biocides, with their activity relying on the electrostatic binding between the positively charged molecules of the biocide and the intrinsically negatively charged cell wall of bacteria, which results in the damage of the latter and subsequently cells death. A breakthrough in the field of contact-killing would be if the bacteria themselves triggered the killing process. It is known that the capacity of bacteria to secrete a series of extracellular metabolites like enzymes and exotoxins accounts for their pathogenicity. For instance, *Pseudomonas aeruginosa*, a pathogen responsible for most urinary tract infections, produces a toxin called pyocyanin. The reduced form of pyocyanin is a highly reactive free radical, which readily reacts with oxygen-producing reactive oxygen species (ROS) such as the superoxide anion and hydrogen peroxide, which in turn are capable of depolymerizing glycosaminoglycans like hyaluronic acid (HA) (Scheme 1).

Francesko et al. [2] have nicely exploited this property of *P*. *aeruginosa* to prepare multilayer coatings for bacteria biofilm prevention on urinary catheters. They used the layer-by-layer (LbL) assembly of polyelectrolytes to build up a multilayer film consisting of alternate layers of the anionic polyelectrolyte HA and sonochemically processed nanospheres prepared from the cationic polyelectrolyte 6-deoxy-6-(ω-aminoethyl) amino cellulose (AC). 

**Scheme 2:**
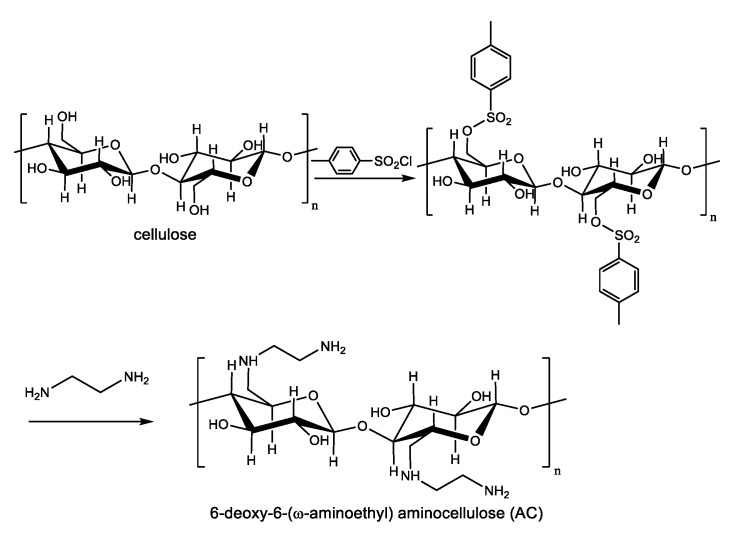
Synthesis of 6-deoxy-6-(ω-aminoethyl) amino cellulose (AC).

The cationic polyelectrolyte AC was synthesized from microcrystalline cellulose as depicted in Scheme 2 through the intermediacy of a tosyl derivative of cellulose [3]. Next, AC nanospheres with a lipid core composed of sunflower oil, were prepared using an adapted sonochemical mediated synthesis previously developed by Suslick [4].

The multilayer coatings were assembled on silicone supports in such a way that the outermost layer, which is in direct contact with bacteria, is the biocidal polycationic layer of AC nanospheres. To this purpose, the silicone support was the first surface-functionalized with amino groups by treatment with 3-(aminopropyl)triethoxysilane (APTES) in order to facilitate the deposition of the first HA layer through electrostatic interactions. Next, the first AC nanospheres layer was deposited, and the procedure was repeated identically until the number of alternate HA/AC bilayers reached the desired value. Pyocianin secreted by *P*. *aeruginosa* destroyed the HA layer between two successive layers of AC nanospheres, thereby releasing the AC nanospheres immediately inward from the outermost layer. Hence, the local concentration of the polycationic antibacterial increased over time following the sequential degradation of each HA layer, which explains the improved antibacterial performance of the (HA/AC nanospheres)_n_ multilayer coating. Moreover, surface nanotopography was characterized by increased roughness due to the presence of massive defects caused by the nanospheres, which in turn facilitated bacterial attachment to the contact-killing surface. Multilayered coatings for which the value of n was as low as 5 were able to prevent the formation of *P*. *aeruginosa* biofilms when incubated with bacteria. In the absence of bacteria, the multilayered coatings were quite stable. This is of notable importance since unnecessary elution and premature depletion of the biocidal agent, namely AC nanospheres from the active nanocoatings, is thereby avoided. Instead, the biocidal AC nanospheres are gradually released from the multi-layered coating following the bacteria-triggered stepwise degradation from the outside inward of the HA component of each HA/AC bilayer. Thanks to this ingenious design, long-lasting (up the seven days) antibiofilm activity was achieved.

### 2.2. Non-Release-Based Antimicrobial Systems (Contact-Killing)

**Scheme 3:**
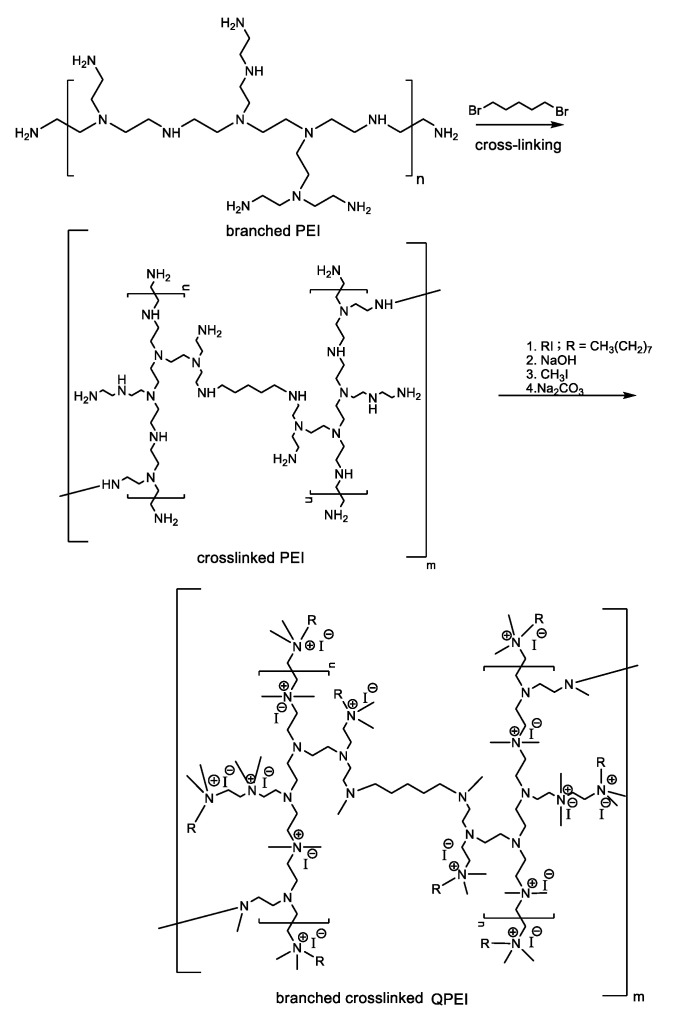
Synthesis of quaternary ammonium branched poly(ethyleneimine) (QPEI) by the *N*-alkylation method.

Biocompatible, non-leachable antimicrobial nanoparticles based on quaternary ammonium branched poly(ethyleneimine) (QPEI) were synthesized and incorporated as an active ingredient in surgical dressing materials for wound healing after maxillectomy [5]. The antibacterial activity of cationic biocides depends on their hydrophobicity and charge density [6]. There are two ways to prepare QPEI: *N*-alkylation and reductive amination. The first method was used by Atar-Froyman et al. [5] who prepared QPEI nanoparticles by crosslinking branched poly(ethyleneimine) (PEI) with 1,5-dibromopentane followed by subsequent alkylation with iodooctane and eventual quaternization with methyl iodide as shown in Scheme 3. PEI is abundant in amino groups facilitating intramolecular crosslinking with 1,5-dibromopentane. Due to the branched structure of PEI, only a small degree of crosslinking is required to obtain insoluble nanoparticles, which is essential for materials such as restorative composites that have to exhibit good resistance to an aqueous environment. *N*-Alkylation of crosslinked PEI with alkyl halides of various chain lengths imparted hydrophobic nature to the resulted nanoparticles, while further treatment with methyl iodide introduced the positively charged quaternary ammonium groups responsible for the biocidal activity. The prepared nanoparticles were incorporated into a commercially available polymethyl methacrylate-based soft liner. The dental wound dressing material with incorporated QPEI nanoparticles showed good biocompatibility both in vitro and in vivo and exhibited strong anti-biofilm effect in vivo inhibiting proliferation of viable *Enterococcus faecalis*, *Streptococcus mutans*, *Staphylococcus aureus*, *P*. *aeruginosa*, *Staphylococcus epidermidis*, and *Candida albicans* strains in post-surgery maxillofacial patients [5].

The second route available for preparing QPEI nanoparticles involves crosslinking of PEI with glutaraldehyde, followed by sequential treatment with octanal and sodium cyanoborohydride (reductive amination) and final quaternization with methyl iodide. Following this method, Azevedo et al. [7] prepared QPEI nanoparticles and tested their capacity to inhibit biofilm development on polyurethane (PUR)-like catheters. At a concentration twice the minimum inhibitory concentration (MIC), the QPEI nanoparticles were able to inhibit biofilm metabolic activity of *S*. *aureus* (2500 mg/L), *S*. *epidermidis* (2500 mg/L), and *A*. *baumannii* (5000 mg/L) [7].

## 3. Antibiofilm Coatings Based on Antimicrobial Peptides (AMPs)

### 3.1. AMPs Releasing Coatings

AMPs are small evolutionally conserved peptides exhibiting bactericidal properties, with the permeabilization of the cellular membrane as the main mechanism of action [8].

Prevention of *S*. *aureus* biomaterial-associated infections was achieved by de Breji et al. [9] through the incorporation of the antimicrobial peptide OP-145 into a polymer-lipid encapsulation matrix (PLEX)-coating. This type of formulation allowed the controlled release of the peptide and the maintenance of high peptide levels at the implant–tissue interphase for prolonged periods of time. The AMP OP-145 is a synthetic derivative of the human cathelicidin LL-37 with the following amino acid sequence: Ile-Gly-Lys-Glu-Phe-Lys-Arg-Ile-Val-Glu-Arg-Ile-Lys-Arg-Phe-Leu-Arg-Glu-Leu-Val-Arg-Pro-Leu-Arg acetylated at the *N* terminus and amidated at the *C* terminus. The lipid-and-polymer-based drug delivery coating (PLEX) consisted of the biocompatible polyester copolymer poly(lactic-*co*-glycolic acid) (PLGA) and the following lipids: Dipalmitoyl phosphatidylcholine (DPPC), distearoyl phosphatidylcholine (DSPC), and cholesterol. The PLEX-coating was prepared by mixing two solutions: One solution containing PLGA and cholesterol dissolved in ethyl acetate (EA) and another solution containing the two phospholipids dissolved in a mixture of methanol and EA [10]. The resulted solution was sprayed on solid medical-grade TAN (titanium 7%-aluminium6%-niobium; ISO 5832/11) intramedullary nails. Residual solvents were removed using evaporation under reduced pressure. To evaluate the antibiofilm activity of AMP OP-145-releasing coatings, the authors focused on some in vivo animal models (mice and rabbits). The intramedullary cavity of rabbits was inoculated with *S*. *aureus,* and subsequently, the animals received an uncoated or a PLEX-OP-145-coated nail. After 28 days, the viable bacteria present on the implants, in the bone cavities, and in the surrounding soft tissues were counted. In rabbits that received implants covered with OP-145-releasing coatings, the contamination ratio of both nails and bones was only 33% as compared with 71% and 100%, respectively, in animals receiving the uncoated nails. Similarly, the ratio of infected soft tissues was only 20% in rabbits with PLEX-OP-145-coated nails, while 71% of the samples taken from rabbits with uncoated nails were culture-positive.

Raman et al. [11] immobilized an antifungal 14-helical β-peptide with high specificity for *Candida albicans* into polymer-based multilayer coatings build-up using the LbL assembly method. Polyelectrolyte multilayer coatings have been constructed on the inner surfaces of flexible catheter tubes made of polyurethane, polyethylene, and silicone. The first deposited layer consisted of the cationic branched PEI. Next, multilayered films were fabricated by alternate deposition of a layer of an anionic polymer (poly-*L*-glutamate (PGA) or HA) and of a layer of a cationic polymer (poly-*L*-lysine (PLL) or chitosan (CHI)) using the deposition technique named “fill-and-purge” [12] so that the ultimate film terminated with an anionic polyelectrolyte. Briefly, the catheter was filled with a solution of the anionic polyelectrolyte PGA or HA and incubated for 3 min or 5 min, respectively. Next, the catheter was rinsed with 0.15 M NaCl aqueous solution to flush out the remaining polymer solution and remove loosely bound polymer. This iterative procedure was repeated identically for the next cationic polyelectrolyte layer (PLL or CHI) and so on. Then, a solution of the β-peptide in 0.15 M NaCl was infused into the catheters coated with (PGA/PLL)_×_(PGA) or (HA/CHI)_×_(HA) multilayers. After 24 h, the peptide solution was removed from the tubes, and the tubes were successively rinsed with phosphate-buffered saline (PBS) for 5 min, a solution of tris(hydroxymethyl)aminomethane-buffered saline Tween (TBST) for 1 h, and again with PBS for 5 min. In vitro biofilm inhibition assays were carried out in synthetic urine (SU) media. Aseptic catheters coated with β-peptide-loaded films as well as control catheters (no-peptide and no-film/no-peptide) were infected by placing 3 cm segments of the catheter tip into suspensions of viable *C*. *albicans* cells in SU and subsequently incubating the tubes at 37 °C for 48 h. At the end of the incubation period, biofilm development was estimated either by performing a metabolic (2,3-bis[2 -methyloxy-4-nitro-5-sulfophenyl]-2*H*-tetrazolium-5-carboxanilide) (XTT) assay or by scanning electron microscopy (SEM) imaging. The results showed that catheters coated with β-peptide-loaded multilayers were able to inhibit the biofilm formation on the intraluminal catheter surfaces.

### 3.2. Contact Active Antimicrobial Surfaces Fabricated by Covalent Immobilization of AMPs

**Scheme 4:**
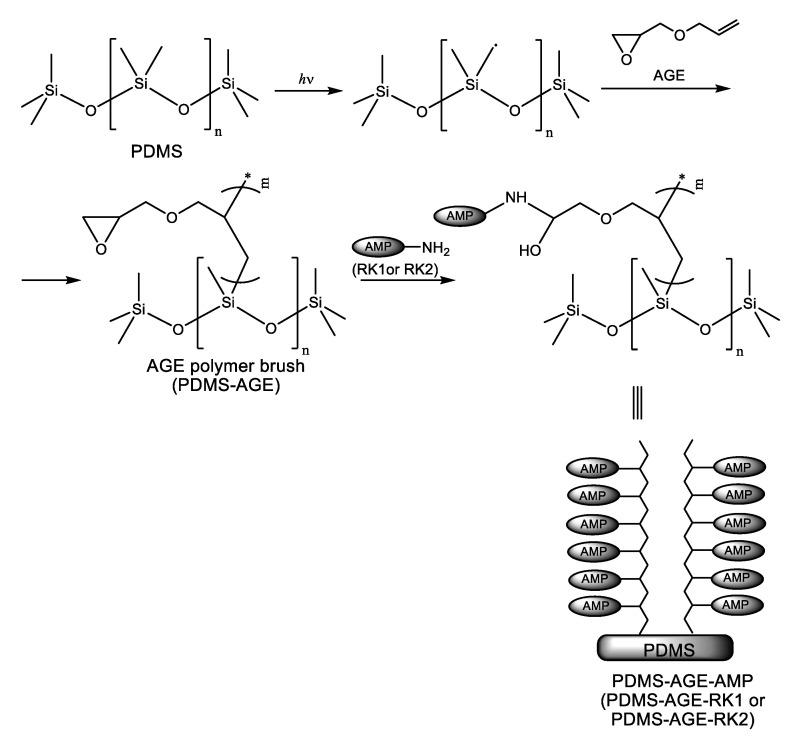
Synthesis of polydimethylsiloxane (PDMS)-allyl glycidyl ether (AGE) polymer brushes and tethering of antimicrobial peptides RK1 and RK2.

Li et al. [13] addressed catheter-associated urinary tract infections (CAUTIs) by covalently immobilizing AMPs on the surface of silicone (polydimethylsiloxane) (PDMS) urinary catheters. The immobilization strategy was based on tethering peptide moieties to an allyl glycidyl ether (AGE) polymer brush synthesized as depicted in Scheme 4. The polymer brush was obtained using the “grafting from” technique. In this polymerization method, the polymerization initiators are immobilized on the surface being subsequently activated or surface-attached reactive species like free radicals, cations, or anions, able to initiate the polymerization reaction are generated in situ through plasma or UV/ozone treatment. In other words, the polymer chains grow from the surface and, therefore, the method is also named surface-initiated polymerization (SIP). Two new short peptides (RK1 and RK2) have been engineered by the authors for enhanced antimicrobial properties and resistance against media with increased salinity, which is thought to reduce the efficacy of the endogenous cationic host defense peptides. The primary structures of these peptides were: Arg-Trp-Lys-Arg-Trp-Trp-Arg-Arg-Lys-Lys (RK1) and Arg-Lys-Lys-Arg-Trp-Trp-Arg-Arg-Lys-Lys (RK2), respectively. Covalent immobilization of RK1 and RK2 endowed PDMS urinary catheter surfaces with antibiofilm properties against *Escherichia coli*, *S*. *aureus,* and *C*. *albicans* in both PBS and urine media. Almost 100% inhibition of bacterial colonization was observed for both bacteria. The immobilized peptides showed no cytotoxicity toward mammalian cells.

**Scheme 5:**
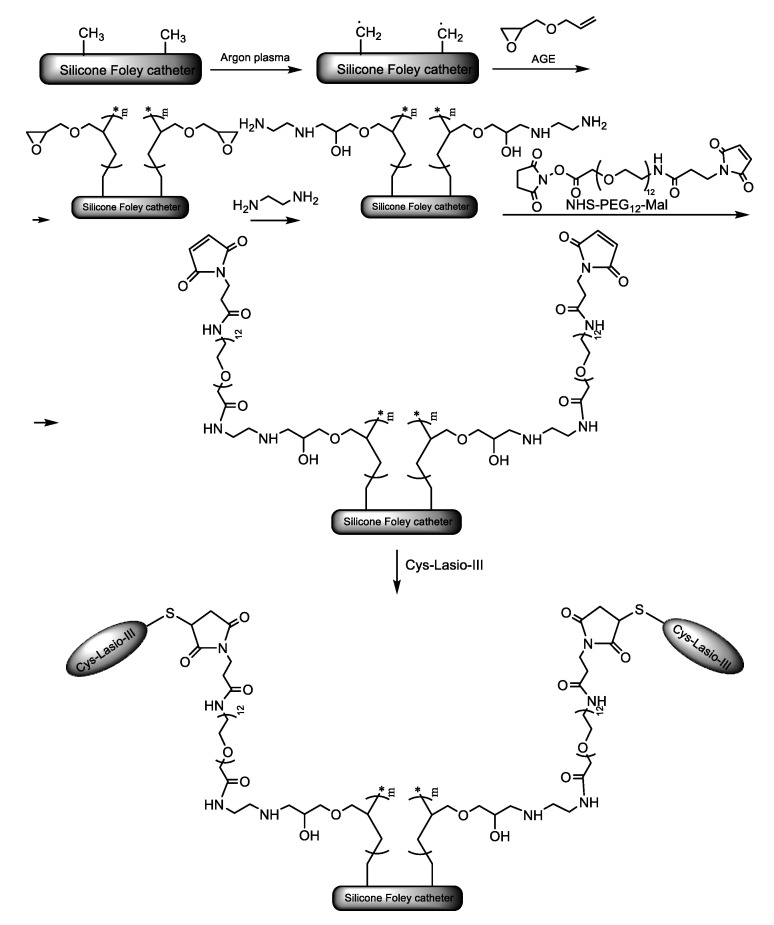
Synthetic strategy to covalently immobilize the antimicrobial peptides (AMP), Cys-Lasio-III, on the surface of silicone Foley catheters [14]. Adapted from an open-access source.

In a closely related approach, Mishra et al. [14] immobilized the potent AMP, Lasioglossin-III (Lasio-III), on silicone Foley catheters. To achieve an effective surface concentration, the authors used a heterotelechelic poly(ethylene glycol) (PEG) spacer to attach the peptide to the PDMS-AGE polymer brush. The spacer had an amine-reactive succinimidyl ester (i.e., *N*-hydroxysuccinimidyl (NHS) ester) at one end and a sulfhydryl-reactive group (i.e., maleimide (Mal)) at the other end. The polymer brush was connected at the NHS-terminal end of the spacer via an ethylenediamine (EDA) linker, while the Mal moiety at the opposite end was used to attach the AMP. To facilitate sulfhydryl coupling, the *N*-terminal end of the peptide chain was chemically modified with a cysteine residue. The whole synthetic pathway is shown in Scheme 5. The peptide immobilized catheters were inoculated with two prevalent bacteria in urinary tract infections, i.e., the Gram-negative *E*. *coli* and the Gram-positive *E*. *faecalis,* and their antibiofilm activity was assessed by XTT and immersion-based killing assays. The AMP coatings on the surface of the catheters reduced the biofilm development by 60% and 40% for *E*. *faecalis* and *E*. *coli*, respectively. Moreover, stability tests, more specific adenosine triphosphate (ATP) leakage assays showed that in SU environment, the catheters with immobilized AMPs preserved their activity against *E*. *coli* cells for at least four days. Furthermore, 3-(4,5-dimethylthiazol-2-yl)-2,5-diphenyltetrazolium bromide (MTT) cytotoxicity assay revealed good biocompatibility of the Cys-Lasio-III-functionalized catheters. The immobilization of the AMP led to a reduced hemolytic effect on human red blood cells (RBC) and THP-1 monocytic cell lines. The development of a urinary catheter that simultaneously shows strong activity against CAUTIs, chemical stability, and good biocompatibility represents a quite remarkable achievement in the field. Using the same synthetic strategy, the AMP WR11 engineered with an *N*-terminal cysteine (CWR11; Cys-Trp-Phe-Trp-Lys-Trp-Trp-Arg-Arg-Arg-Arg-Arg-NH_2_) was immobilized on PDMS slides [15].

**Scheme 6:**
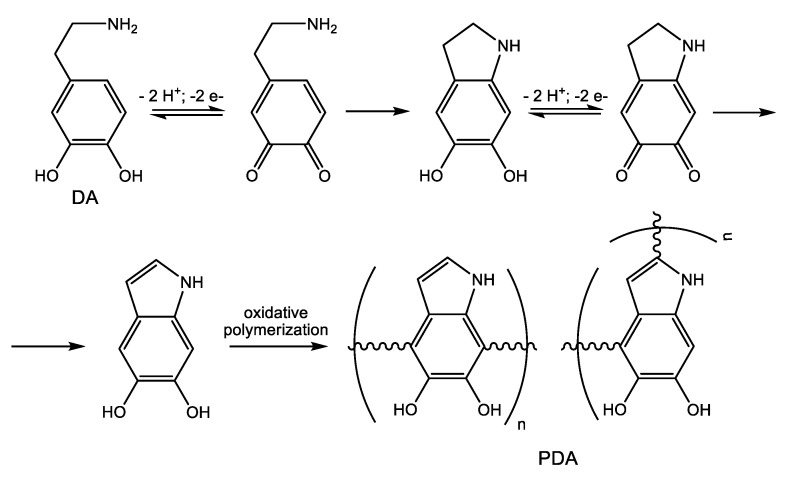
Oxidative self-polymerization of dopamine (DA).

However, the immobilization technique described above suffers from a major drawback, which is the complicated surface activation method (plasma radiation). To overcome this limitation, Lim et al. [16] applied a bioinspired facile conjugation technique previously developed by Messersmith and coworkers [17]. In this immobilization strategy, the synthetic AMP CWR11 was attached to PDMS slides and Foley urinary catheters via an intermediate crosslinking mussel-inspired [18] polydopamine (PDA) film. It is well known that under weak alkaline conditions, dopamine (DA) undergoes oxidative self-polymerization [19,20] (Scheme 6), forming PDA, a soft and sticky material able to adhere to almost any surface. The oxidative polymerization of DA involves catechol-quinone conversions, thereby facilitating covalent fixation of CWR11 either via Michael addition reactions of the biological amino and sulfhydryl nucleophiles in the peptide to the conjugated quinone systems or through the formation of Schiff bases. Briefly, commercially available Foley urinary catheters were covered with a layer of PDA using the dip-coating technique. The catheters were dipped in a solution of DA in 50 mM tris(hydroxymethyl)aminomethane-hydrochloride (TRIS) buffer (pH = 8.8), and the polymerization reaction was allowed to proceed for 72 h at room temperature. Next, the catheters were repeatedly rinsed with deionized water to remove any traces of unattached DA, and then were immersed for another 72 h in a solution of the CWR11 peptide in TRIS buffer at room temperature. Eventually, the peptide-immobilized catheters (Cat-PDA-CWR11) were thoroughly washed with water. The antibiofilm properties of Cat-PDA-CWR11 were assessed in comparison to bare catheter controls. The catheters were seeded into the intraluminal space with equal amounts (50 μL) of suspensions of the most relevant urinary tract infections bacteria (*E*. *coli*, *S*. *aureus*, and green fluorescent protein (GFP)-*P*. *aeruginosa*) each of the same concentration (5 × 10^4^ colony forming units (CFU), incubated for 3 h at room temperature, and then immersed in fresh medium for overnight culture. Bacterial adhesion to catheters was quantitatively assessed by fluorescence spectroscopy. There was up to 92% decrease in the amount of viable adherent (GFP)-*P*. *aeruginosa* bacteria on Cat-PDA-CWR11 as compared to control (unmodified) catheters. Bacterial growth in the immersion medium was monitored by measuring the optical density at 600 nm (OD_600_). For the CWR11-functionalized catheters, no bacterial growth could be detected, whereas significant bacterial growth was evident in the control catheters. The stability of the functionalized catheters was tested by exposing the catheters for three days to various media (air, water, PBS, SU, and urine) and then assessing their antibacterial activity. The Cat-PDA-CWR11 maintained their antibacterial activity in all tested media. In water, the antibacterial activity of CWR11-functionalized catheters was kept unchanged up to 21 days.

To assess the hemolytic activity of the Cat-PDA-CWR11 against RBC, the catheters were immersed in an erythrocyte solution, incubated at 37 °C for 1 h, and vigorously stirred. After centrifugation of the RBC solutions, the hemoglobin released into the supernatant was determined by measuring the optical density at 540 nm. The determined hemolytic activity corresponded to a level of acceptable amounts of hemolysis, as stipulated by ISO 10993 for medical devices.

## 4. Antibiofilm Coatings Based on Antimicrobial Enzymes

Antimicrobial enzymes emerged as a very attractive bioinspired antibiofilm strategy and as a very promising alternative to the classical antibiotic approach since these enzymes widespread among living things act as a defense mechanism against infection from microorganisms being able to directly attack bacteria, interfere with biofilm formation, and/or catalyze reactions that generate antimicrobial compounds.

**Scheme 7:**
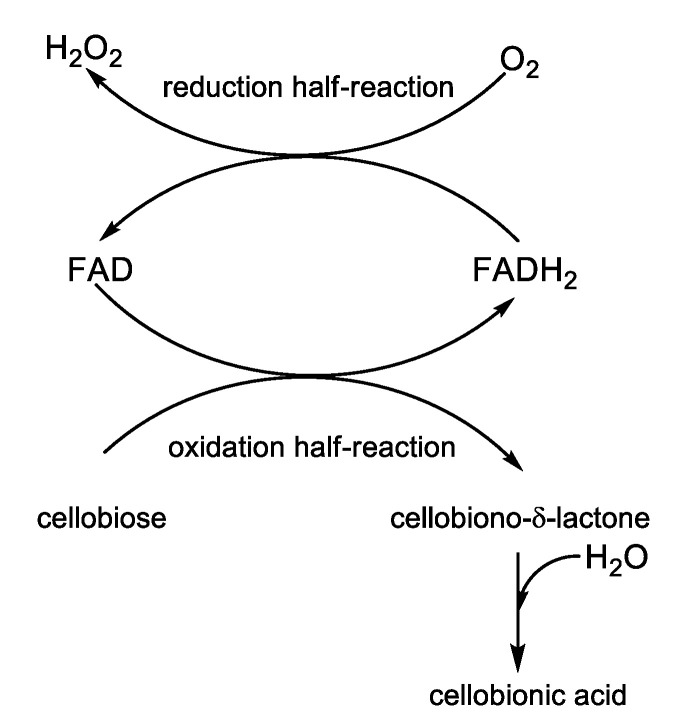
Mechanisms of action of the flavocytochrome cellobiose dehydrogenase.

Many enzymes from different classes exhibit antimicrobial activity [21]:

(i) Proteolytic enzymes
  - Subtilisins that hydrolyze adhesins thereby impeding bacterial attachment onto solid supports;
  - Lysostaphin, which cleaves the pentaglycine bridges of *Staphylococci* cell;
  - Bacteriophage lysins, which are peptidoglycan hydrolases that cause osmotic lysis and cell death of the bacterium;
(ii) Polysaccharide degrading enzymes
  - Lysozyme, which catalyzes the hydrolysis of the glycosidic bond between *N*-acetylmuramic acid and *N*-acetyl-*D*-glucosamine residues in peptidoglycan thereby damaging the bacterial cell wall;
  - Alpha-amylases;
  - Dispersin B (DspB), which disrupts biofilm formation by catalyzing the hydrolysis of the β-1,6-glycosidic linkages within poly-β-1,6-N-acetyl-D-glucosamine, which is an important polymer needed for attachment of *E*. *coli* and *Staphylococcus epidermidis* biofilms onto surfaces.
  - Alginate lyase;
(iii) Oxidative enzymes
  - The hydrogen peroxide producing enzymes like glucose oxidase (GOx) and cellobiose dehydrogenase (CDH); cellobiose is a reducing disaccharide, which donates electrons getting oxidized to cellobiono-δ-lactone, while electrons are accepted by oxygen, which in turn is reduced to hydrogen peroxide (see the redox half-reaction in Scheme 7); the known disinfectant and antiseptic properties of hydrogen peroxide rely on its capacity to generate free radicals able to attack lipid membranes, DNA, and other vital constituents of the bacterial cell;
  - Hydrogen peroxide using enzymes like haloperoxidases, lactoperoxidase, and myeloperoxidase, which catalyze the oxidation of halide and thiocyanate anions to more potent antimicrobial agents consuming the hydrogen peroxide produced by the hydrogen peroxide producing enzymes;
(iv) Quorum-quenching enzymes like lactonases, which catalyze the hydrolysis of the ester bond of acylated homoserine lactones thereby impeding the binding of these autoinducers to their target transcriptional regulators;
(v) DNases that cleave DNA by catalyzing the hydrolysis of the phosphodiester bonds of the sugar-phosphate backbone; e-DNA derived from lysed bacteria play an important role in the initial establishment of the biofilm and subsequently in the maintenance of the biofilm’s structural integrity acting as an effective crosslinker.

CDH was covalently immobilized on PDMS following a four-step synthetic pathway developed by Thallinger et al. [22]. First, PDMS urinary catheters were washed with 96% ethanol and distilled water, and next were subjected to a low-pressure oxygen plasma treatment in order to obtain hydroxy-functionalized PDMS surface. The second step of the functionalization process consisted of the silanization with (3-aminopropyl)-triethoxysilane (APTES) to yield an amine-modified PDMS surface. In the third step, the homobifunctional linker glutaraldehyde (GA) was tethered to the amine-terminated PDMS surface through one of the terminal aldehyde functions. The aldehyde group left free at the other end of the linker was used to covalently attach the enzyme in the fourth step of the synthesis. The surface immobilization did not affect the activity of CDH, as it was demonstrated by measuring the amount of hydrogen peroxide produced by the immobilized enzyme. The enzymatic activity was determined in buffered media of different pH values (5.5; 6.0; 7.3; 8.0). The best enzymatic activity was obtained at pH = 8.0. To assess the effect of SU on the activity and long-term stability of CDH, CDH-functionalized PDMS samples were incubated in SU at 37 °C for 16 days, and the residual activity was measured every two days. No enzyme leaching was detected, but the enzyme activity showed a remarkable decay (about 40%) during the first two days, followed by a much slighter decrease until day 16 when the enzyme activity reached 20% of the initial value. The antibiofilm activity of immobilized CDH was evaluated in vitro under dynamic conditions using an artificial bladder system model. CDH-coated Foley catheters and ordinary catheters were inoculated with *S*. *aureus* (OD_600_ = 0.01). After seven days, different segments of the catheters were cut and analyzed for biofilm development. Using the live/dead double staining protocol, viable cells and dead cells were monitored simultaneously with a fluorescence microscope. The surface of the balloon of pristine PDMS catheters was completely covered by viable *S*. *aureus* biofilm, while only a few dead populations of *S*. *aureus* could be observed in CDH-functionalized catheters. The total biomass formed on the catheter balloon was quantified using the crystal violet assay. The principle of the method consists of the binding of the cationic dye to the negatively charged DNA and polysaccharide molecules in the extracellular biofilm matrix. The respective catheter segment was dried and stained with crystal violet. After washing with water, the bound crystal violet was redissolved in acetic acid, and the absorbance of the solution was read at 595 nm. As compared to bare silicone catheter controls, the total biomass on the catheter balloon of CDH-coated PDMS catheters was reduced by 70%. The CDH-coated silicone catheters exhibited good biocompatibility, as shown by the MTT cell viability assays on human embryonic kidney cells (HEK 239) and mouse macrophages (RAW 246,7).

**Scheme 8:**
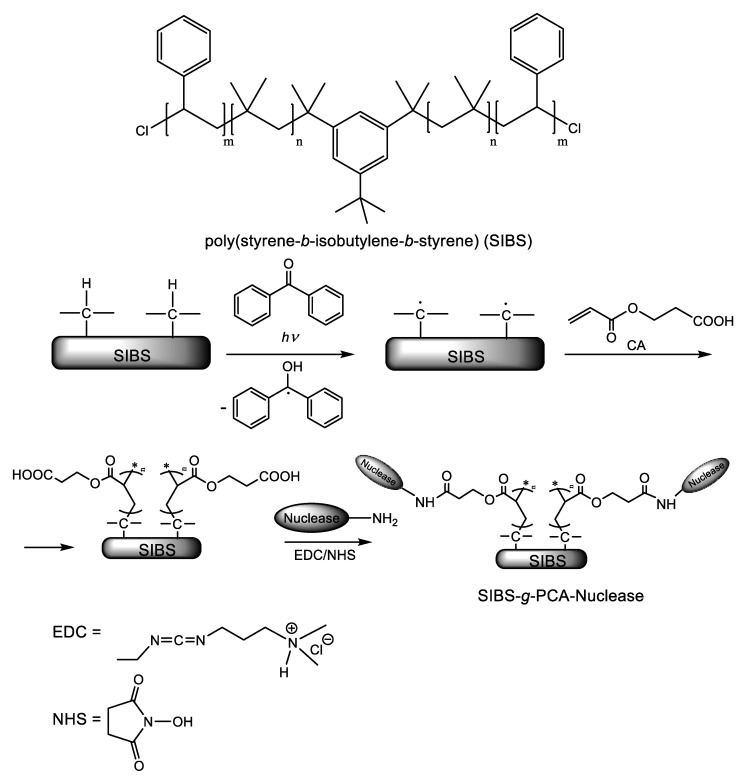
The structure of the biostable thermoplastic elastomer poly(styrene-*b*-isobutylene-*b*-styrene) (SIBS) and the synthetic pathway to SIBS-*g*-PCA-Nucleases [23]. Adapted with permission from Nuclease-Functionalized Poly(Styrene-b-isobutylene-b-styrene) Surface with Anti-Infection and Tissue Integration Bifunctions. Copyright (2014) American Chemical Society.

Surface-immobilization of deoxyribonuclease I (DNase I) and ribonuclease A (RNase A) on the tri-block thermoplastic elastomer poly(styrene-*b*-isobutylene-*b*-styrene) (SIBS) was achieved by Yuan et al. [23] using a “grafting from” strategy. Benzophenone (BP) was used as a photoinitiator for the SIP of 2-carboxyethyl acrylate (CA), as illustrated in Scheme 8. The pendant carboxyl groups of the resulted polymer brush were used as anchoring points for the subsequent attachment of the nucleases through the well-documented amide bond formation (1-ethyl-3-(3-dimethylaminopropyl)carbodiimide (EDC) hydrochloride/NHS) strategy. To assess the antibiofilm activity, the SIBS substrates modified with nucleases were incubated in a growth medium containing 10^8^ bacterial cells (*E*. *coli* and *S*. *aureus*)/mL for 10 h at 37 °C. After decantation of the growth medium and rinsing with PBS to remove any planktonic bacteria, nuclease-modified SIBS samples were subjected to a live/dead cell double staining assay using a mixture of the membrane-permeable green fluorescent nucleic acid dye SYTO 9 and membrane-impermeant red-fluorescent nucleic acid dye propidium iodide (PI). Observing the samples under laser scanning confocal fluorescence microscopy, the viable cells (appearing green) could be distinguished from the dead cells (appearing red).

The antibiofilm activity was evaluated by counting the percentage of the area occupied by the bacterial biofilm. Some differences between the two nucleases could be observed. In the case of DNase, the substratum surface covered by the *E*. *coli* biofilm was decreased from 88% for the bare SIBS control to only 0.3% for the SIBS-*g*-PCA-DNase, while the *S*. *aureus* biofilm was reduced from 68.1% of the pristine (unfunctionalized) SIBS support to 2.6% of SIBS-*g*-PCA-DNase. However, the inactivation of DNase coating resulted in a dramatic increase in the level of substratum coverage by *E*. *coli* biofilm. RNase-functionalized SIBS substrates were proved to be more effective against *E*. *coli* (proportion of the substratum surface contaminated with biofilm bacteria equals about 1.5%) than against *S*. *aureus* (21.4% of the substratum area was covered by bacterial biofilm). Unlike DNase-functionalized SIBS, the antibacterial activity of SIBS-*g*-PCA-RNase was independent of nuclease activity, proving that the capacity of SIBS-*g*-PCA-RNase to inhibit bacterial adhesion was mainly due to its hydrophilicity. Furthermore, the authors tested the capacity of these nuclease coatings to reduce the interactions of host proteins with the surface of SIBS medical devices, thereby avoiding the formation of a conditioning film and further bacterial colonization. Investigating the adsorption of bovine serum fibrinogen (BFg), the authors found out that the amount of adsorbed Bfg was reduced from 6.5 μg/cm^2^ for unfunctionalized SIBS to about 1.8 μg/cm^2^ for SIBS-*g*-PCA-DNase and to less than 1.5 μg/cm^2^ for SIBS-*g*-PCA-RNase.

Using a synthetic strategy, Swartjes et al. [24] immobilized DNase I on polymethylmethacrylate (PMMA) substrate via a dopamine intermediate coupling layer. Initial bacterial adhesion to the DNase I-coated PMMA substrate was strongly inhibited compared to inactive control substrates, namely bare PMMA, dopamine-coated PMMA, and heat-inactivated DNase I-coated PMMA. The bacterial cells adhered to the substrate surfaces during the first 60 min were counted using fluorescence microscopy after staining the biofilm slime with live/dead stain (BacLight™ Bacterial Viability and Counting Kit). Coating of the PMMA substrate with active DNase I produced a reduction of the number of adhering bacteria by 99% for *P*. *aeruginosa* PAO1 and 95% for *S*. *aureus* ATCC 12600 compared to controls. Moreover, biofilm growth was monitored by confocal laser scanning microscopy (CLSM). CLSM images of biofilms developed on the substrata showed average biofilm thicknesses of 10 μm and only of 0.2 μm for *P*. *aeruginosa* PAO1 biofilms grown on inactive control substrata and on DNase I-coated PMMA substrate, respectively. Similarly, in the case of *S*. *aureus* ATCC 12600, biofilm thickness was reduced from 18 μm for controls to 3 μm for the active DNase I coating. Biocompatibility of DNase I coatings with mammalian cells was assessed in vitro using human osteosarcoma U2OS cells. Neither adhesion nor proliferation of U2OS cells onto DNase I coated PMMA substrate was affected.

## 5. Coating Materials with Embedded Antibiotics and Other Antibiofilm Agents

### 5.1. Antibiotic Based Antibiofilm Coatings

Both controlled drug release from coatings containing physically entrapped antibiotics and covalent immobilization of antibiotics onto solid surfaces have been taken into account as active antibiofilm strategies.

Jennings et al. [25] developed a novel antibiotic eluting material based on phosphatidylcholine that could be applied as an implant coating at point of care. The lipid matrix was loaded with 25% antibiotic (amikacin (A) or vancomycin (V) or a mixture of 12.5% of both antibiotics (AV)). In vivo biofilm inhibition was studied in a mouse model of implant-associated infections. Ten catheters have been modified to include a stainless-steel wire, which either has been subsequently covered by a thin film coating with and without embedded antibiotics or remained uncoated. The catheters were divided into five experimental sets, each set comprising two catheters. The two catheters in each set were implanted subcutaneously in dorsal flank regions of mice, bilaterally, one each on the right and left side of the spine. After implantation, the catheter segments were infected by the intraluminal inoculation of a polymicrobial mixture of 10^5^ CFU of *S*. *aureus* and 10^4^ CFU of *P*. *aeruginosa*. The following modified catheters differing by the sort of the inserted stainless-steel wires were used: Control (uncoated wires, U), U wires, the antibiotics (A and V) being co-administered to the mice via injection, phosphatidylcholine-coated wires (P), AV-coated wires, A-coated wires, and V-coated wires. After 48 h from implantation, the catheters and wires were removed from the mice, rinsed, and SEM images were taken for visual analysis of attached bacterial colonies. The wires with antibiotic-loaded coatings showed 100% clearance rate from *S*. *aureus* and a 90% clearance rate of *P*. *aeruginosa*.

To prevent implant-associated osteomyelitis caused by methicillin-resistant *S*. *aureus* (MRSA), the group of Moriarty [26] used the polymer-lipid encapsulation matrix (PLEX) as a nanoplatform for controlled release local delivery of doxycycline (DOX). The PLEX platform was prepared as previously described in Section 3.1. The doxycycline release profile was characterized by an initial burst effect, 25% of the antibiotic-loaded being released within the first 24 h, followed by zero-order release kinetics for the next 27 days. The efficacy of the biodegradable PLEX-doxycycline implants was evaluated in a rabbit intramedullary nail model. Sixteen rabbits were allocated into two groups of eight animals each. A volume of 100 μL (4.7 ± 1.2 × 10^5^ CFU) of MRSA strain LUH15101 resistant to penicillin, oxacillin, tetracycline, doxycycline, and cotrimoxazole was inoculated into the medullary cavity of the humerus. The animals in the control group received an uncoated intramedullary nail manufactured from medical grade TAN, while the other eight animals received a doxycycline-containing PLEX-coated nail. After four weeks, all the rabbits in the control group showed culture-positive implants and bone, whereas in the PLEX-doxycycline coated group, 57% of the animals were free from MRSA implant and bone infections.

**Figure 1:**
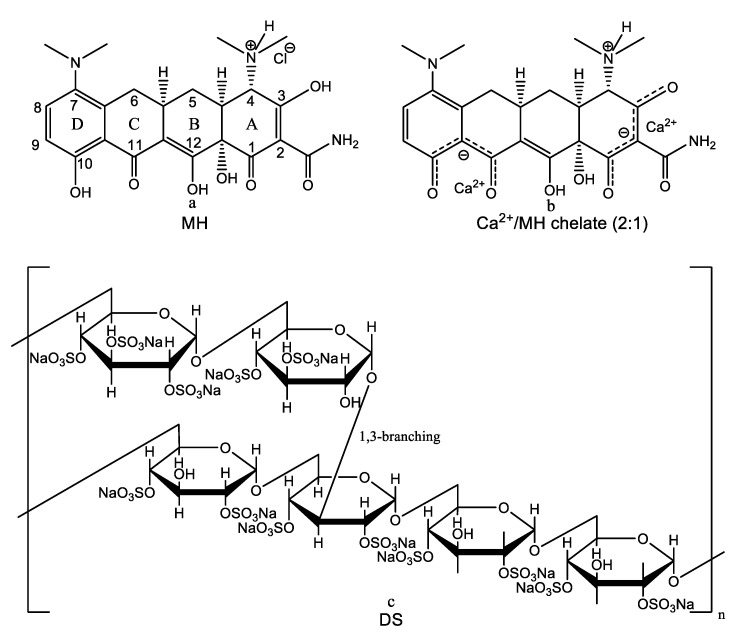
Chemical structures of minocycline hydrochloride (**a**), 2:1 Ca^2+^/minocycline hydrochloride chelate (**b**), and dextran sulfate sodium salt (**c**).

Hydrophilic coatings are known to prevent nonspecific protein adsorption and subsequent bacterial colonization [27]. If the antifouling properties could be combined with the controlled release of an antimicrobial agent, the antibiofilm efficacy of these coatings would be greatly increased. However, this approach is hampered by the fact that most water-soluble low molecular weight antimicrobial agents diffuse very fast out of these hydrophilic coatings. Zhang et al. [28] came up with an ingenious solution to the above problem making use of a different loading and release mechanism. They achieved the sustained release of minocycline hydrochloride (MH), a broad-spectrum antibiotic, and anti-inflammatory drug belonging to the tetracycline family, from hydrophilic multilayer coatings prepared by the LbL self-assembly technique. The active antibiotic ingredient MH was loaded into the polyelectrolyte multilayer film as one of the multilayer components. As a tetracycline derivative, MH has a strong tendency to chelate Ca^2+^ ions. Chelation sites include the enol system in positions 1 and 3 of the ring A, the carboxamide function (Position 2), as well as the β-diketone system present at positions 11 and 12 (Figure 1a) [29]. One molecule of MH can chelate one or two Ca^2+^ ions (Figure 1b) [30]. Dextran sulfate (sodium salt) was used as anionic polyelectrolyte (Figure 1c), while the cationic polyelectrolyte was gelatin type A (GA), a biodegradable natural polymer derived from collagen that is positively charged at physiologically relevant pH values. The self-assembled multilayer coatings were deposited on UV-transparent or black 96-well plates and on silicon wafers, the substrates being previously coated with an initiating positively charged base layer of PEI. The deposition was achieved simply by alternately dipping the substrate into solutions of DS, MH, and GA with or without Ca^2+^ for 10 min and rinsing with deionized water between dipping steps. The LbL multilayer film growth and incorporation of MH and GA in the film were monitored by UV-vis spectroscopy and fluorescence spectroscopy, respectively. The incorporation of MH in the LbL coating was confirmed or infirmed spectrophotometrically by detecting the characteristic absorbance marker peak at 245 nm (DS and GA have negligible absorbance at this wavelength). Similarly, GA incorporation was evidenced by measuring the fluorescent intensity of the fluorescein isothiocyanate (FTIC)-labeled GA. Interestingly, the authors found out that the formation of the LbL assembly and MH incorporation into the multilayered coatings crucially depend on the presence of Ca^2+^ ions in the DS solution. This conclusion relies on the following experimental outcomes: (i) Negatively charged DS and positively charged MH-Ca^2+^ chelates failed to form bilayer LbL assembly; (ii) on the other hand, when Ca^2+^ ions were added to the DS solution, the LbL assembly was formed regardless of the presence or absence of Ca^2+^ ions in the MH solution; (iii) although multilayered films of DS/GA could be successfully constructed, MH incorporation into the hydrophilic polyelectrolyte multilayer was not achieved in the absence of Ca^2+^ from the DS solution. The authors rationalized these data concluding that MH incorporation is not ruled by electrostatic interactions but rather by a different mechanism mediated by calcium-binding. By chelating two Ca^2+^ ions, MH acted as an effective crosslinker to bind Ca^2+^ ions in adjacent DS layers, thereby promoting LbL assembly. Moreover, this calcium binding-mediated loading mechanism provided an opportunity to achieve the “on-demand” release of the loaded antibiotic. The chelating ability is pH-dependent, i.e., the Ca^2+^ binding affinity of MH decreases with decreasing pH. Hence, if the surrounding medium becomes more acidic, the chelation between MH and DS-bound Ca^2+^ is weakened, facilitating MH release. Because acidic pH predominates at inflammatory loci and infection sites, this polyelectrolyte multilayer nanoplatform provides a pH-triggered smart drug release system for targeted delivery of MH. In order to optimize the performances of the fabricated hydrophilic multilayer coatings in terms of loading capacity and sustained release the authors studied: (i) The effect of the concentration of Ca^2+^ ions in the coating solutions on MH loading and release, (ii) the effect produced by incorporation of Ca^2+^ ions into MH and GA layers on MH release kinetics, and (iii) the effect of the numbers of DS+Ca^2+^/MH/GA and DS+Ca^2+^/MH+Ca^2+^/GA+Ca^2+^ trilayers on MH release. The optimal configuration that ensures the highest drug density of 645 μg/mm^3^ and the best release kinetics characterized by a low initial burst effect followed by a slow, stable release for over 35 days was found to be (DS+Ca^2+^/MH+Ca^2+^/GA+Ca^2+^)_8_ and was prepared at the optimal concentration of 7.2 mM Ca^2+^ in the coating solutions. The thickness of (DS+Ca^2+^/MH+Ca^2+^/GA+Ca^2+^)_8_ film was 402 ± 19 nm corresponding to an average trilayer thickness of 50 nm. After drug release, the thickness of the film was decreased only by about 100 nm, indicating that MH release from the LbL coating was not due to the degradation of the LbL film. The antibiofilm activity of the (DS+Ca^2+^/MH+Ca^2+^/GA+Ca^2+^)_8_ film was tested against several bacterial strains including *E*. *coli* ATCC 25922, *E*. *coli* 0157:H7, *Acinetobacter baumannii* ATCC 19606, *S*. *aureus* ATCC 25923, MRSA USA300, *S*. *epidermidis* ATCC 12228, and a clinical isolate *A*. *baumannii* by comparison with bare polystyrene substrate and substrate coated with a (DS+Ca^2+^/GA+Ca^2+^)_8_ film. The XTT assays demonstrated that coatings with incorporated MH inactivated bacteria in all tested biofilms. Furthermore, a live/dead cell double staining assay using SYTO 9 and propidium iodide dyes revealed complete elimination of multi-drug resistant *A*. *baumannii* biofilm on coatings loaded with MH.

Another strategy that exploits variations in pH to control the loading and release of antimicrobial compounds consists of the development of polyelectrolyte multilayer microcapsules with pH-tunable permeability [31,32,33,34,35]. Anandhakumar et al. [35] accomplished pH-controlled ciprofloxacin hydrochloride (CH) encapsulation in and release from polyelectrolyte multilayer microcapsules fabricated by LbL deposition of poly(allylamine hydrochloride) (PAH) and poly(methacrylic acid) (PMA) on poly(styrene sulfonate) (PSS)-doped calcium carbonate particles as sacrificial templates. Spherical PSS doped CaCO_3_ microparticles with a diameter of 6 ± 0.5 μm were prepared by co-precipitation of CaCl_2_ and Na_2_CO_3_. The dopant PSS was dissolved in the Na_2_CO_3_ solution prior to mixing equal amounts of the two solutions [33,34,35]. Since the CaCO_3_ microparticles have been synthesized in the presence of the anionic polyelectrolyte PSS, their surface was negatively charged, as evidenced by zeta potential measurement [33]. Hence, the first layer deposited on the surface of the CaCO_3_ microspheres templates was the positively charged polyelectrolyte PAH followed by deposition of the oppositely charged polyelectrolyte PMA and the iterative procedure was repeated until four PAH/PMA bilayers were deposited. The negatively charged PSS molecules adsorbed on the surface of the template also play the role of preventing the recrystallization of CaCO_3_ into the more stable rhombohedral calcite polymorph [34]. CaCO_3_ particles have been chosen to template the synthesis of the polyelectrolyte multilayer microcapsules because they can be easily removed with a chelating agent like ethylenediaminetetraacetic acid (EDTA). After the deposition of the polyelectrolyte multilayers was completed, the calcium carbonate core was dissolved by 0.2 M EDTA solution [35]. During this process, the negatively charged PSS molecules are entrapped inside the microcapsules imparting them with the capability to spontaneously load positively charged antimicrobial compounds like CH [33,35]. The pH-tunable permeability of the walls of the microcapsules was evidenced by monitoring the incorporation of the fluorescent-labeled positively charged tetramethylrhodamine isothiocyanate (TRITC)-dextran polymer at different pH values. In alkaline media, e.g., pH = 8.0, the interior of the capsules remained dark, indicating that no loading of TRITC-dextran occurred. One says that the microcapsule is in its “closed” state. Under acidic conditions (pH = 3.0), strong fluorescence was observed, proving that a large amount of TRITC-dextran has penetrated the capsules walls getting inside the capsules [35]. Hence, the microcapsule state was switched from the “closed” state in alkaline conditions to the “open” state in acidic media. This was rationalized as follows: By decreasing the pH, more and more carboxylate groups of the PMA are protonated; hence, the positively charged ammonium groups in PAH remain uncompensated thereby developing an electrostatic repulsive force resulting in phase segregation and promoting the transition from a continuous to nanoporous morphology of the capsule walls [32,34]. The excellent loading capacity for CH of PAH/PMA multilayer microcapsules (236 mg/mL) in acidic media (pH = 3.0) was attributed to two main reasons: (i) The higher degree of protonation of the permeant at pH = 3.0, and (ii) the increased permeability of the walls of the microcapsules in acidic media favoring the diffusion of the positively charged antibiotic molecules from the bulk into the interior of the capsules preloaded with the negatively charged polyelectrolyte PSS. The release of CH from PAH/PMA microcapsules was investigated in PBS (pH = 7.4). The release process was governed by a concentration-dependent diffusion mechanism. The release profile is characterized by an initial burst effect during the first 1 h, followed by sustained release. About 70% of the loaded antibiotic was released within 6 h; beyond this period of time, the release curve reached a plateau. The drug release rate could be further slowed down by crosslinking the wall components of the polyelectrolyte capsules. Crosslinking was achieved by incubating CH loaded microcapsules with 0.2 mM EDC at pH = 6.0, at room temperature [35]. The antibacterial efficiency was estimated by adding the bacterial pathogen *E*. *coli* at an infective dose 1.2 × 10^7^ CFU/mL to a suspension of CH loaded crosslinked PAH/PMA polyelectrolyte multilayer microcapsules and monitoring bacterial growth over time. Due to the sustained release of CH from the capsules, no bacterial proliferation was observed up to 10 h [35].

Neut and coworkers [36] introduced a novel and robust gentamicin loaded hydroxyapatite (HAP) coating on titanium alloy (Ti-6Al-4V) covered with a protective, biodegradable PLGA overlayer. HAP was spray-coated on grit-blasted Ti-6Al-4V, according to DePuy’s standard specification for the clinically established Corail^®^ total hip system. Next, gentamicin sulfate was sprayed on HAP-coated, grit-blasted Ti-6Al-4V coupons until the final concentration of 1mg active ingredient/cm^2^ was reached. Eventually, a PLGA layer was deposited over the HAP-containing gentamicin coating using an ultrasonic coating process. The biodegradable protective PLGA overlayer prevents the rapid washout of the gentamicin layer by body fluids rather than controlling the antibiotic release. PLGA-gentamicin-HAP coatings showed an initial burst release of about 95% of the incorporated antibiotic during the first 1–2 h, which is very important since it immediately provides a very high local antibiotic concentration that kills bacteria introduced during the surgery and prevents biofilm development. The initial burst release was followed by slow release up to seven days [36]. In vitro antibacterial efficacy, assays showed that PLGA-gentamicin-HAP coatings killed even gentamicin-resistant *S. epidermidis* 7388 and *S. epidermidis* 7391 strains [36]. Moreover, in a rabbit model of antibiotic prophylaxis for bacterial infection prevention, HAP- and PLGA-gentamicin-HAP-coated pins, respectively, were inserted in bacterially contaminated medullary canals of the animals. The number of staphylococci recovered from femora and the pin itself deriving from the animals that received a PLGA-gentamicin-HAP-coated pin was several orders of magnitude lower than the one corresponding to the animals that received a HAP-coated pin at day 2 and 7 after insertion [36].

Recently, our research group reported the successful matrix-assisted pulsed laser evaporation (MAPLE) deposition of thin coatings consisting of mesoporous silica nanoparticles (MSNs) loaded with the advanced generation cephalosporin antibiotic ceftaroline fosamil or Zinforo (ZiN) on several types of substrates (double side polish silicon, glass, and commercial pure Ti grade 2 disks) [37]. Most important, the MAPLE-deposited ZiN-loaded mesoporous silica nanocoatings were able to release the antibiotic in bioactive form. The MSNs were prepared by the classical base-catalyzed sol-gel method in the presence of cetyltrimethylammonium bromide (CTAB) as a sacrificial template. The MSNs were loaded with the antibiotic by mixing in a grinding mortar with a chloroform dispersion of ZiN until complete evaporation of the solvent. The deposition process was conducted with various laser fluences (300, 400, and 500 mJ/cm^2^), the best compromise between the deposition rate and the stoichiometric transfer being obtained for a fluence of 500 mJ/cm^2^. The biocompatibility of the fabricated nanocoatings was proved both in vitro on cultured human cells, and in vivo using a mouse model. The antibiofilm activity was assessed by a viable cell count (VCC) assay. The bacterial inoculum was prepared by adding 20 μL of an *E*. *coli* ATCC 25922 bacterial suspension adjusted to 0.5 McFarland standard to 2 mL of nutritive broth. After inoculation, the tested substrates (glass substrates coated with MAPLE-deposited ZiN-loaded mesoporous silica nanofilm) and the control substrates (bare glass substrates) were incubated at 37 °C for 24 h. Next, the substrates with attached bacteria were washed with PBS, transferred in a fresh well containing 2mL sterile nutritive broth, and incubated for various periods of time (24, 48, and 72 h). At the end of the incubation periods, the biofilm developed was detached from the substrate by vigorous vortexing. After serial dilutions, the PBS suspensions of the detached bacteria cells were seeded on nutritive agar. The bacterial cell concentration in suspension (CFU/mL) was estimated from optical density measurements. The outcomes of the assays showed that the fabricated antibiotic containing coatings exhibited strong antibiofilm activity against *E*. *coli* during the early stage of biofilm formation, i.e., at 24 h. The antibiofilm activity corroborated with the excellent biocompatibility and biodistribution of the prepared nanocoatings recommends them as efficient drug delivery systems with potential applications in bone implantology.

He et al. [38] reported surface functionalization of titanium substrates with the third-generation cephalosporin antibiotic cefotaxime (sodium salt; CS). They used PDA as an adhesive primer for anchoring the antibiotic to the surface of the metal substrate. The catechol/quinone groups in PDA were used as robust anchor points for further tethering of CS. Biofilm formation on CS-decorated Ti was estimated by a double staining assay using the fluorescent staining agents SYTO 9 and propidium iodide. Confocal laser scanning microscope (CLSM) images taken after seven days of incubation with *E*. *coli* and *Streptococcus mutans* showed no trace of biofilm on the CS-decorated Ti interface, whereas extensive *E*. *coli* and *S*. *mutans* biofilm reaching about 30 μm thickness colonized the pristine Ti interfaces. Moreover, the CS-functionalized interfaces showed good biocompatibility and did not cause any significant hemolysis. The authors concluded that the modified Ti substrate is a promising candidate among possible dental and bone implant materials.

### 5.2. N-halamine Compounds

**Figure 2:**
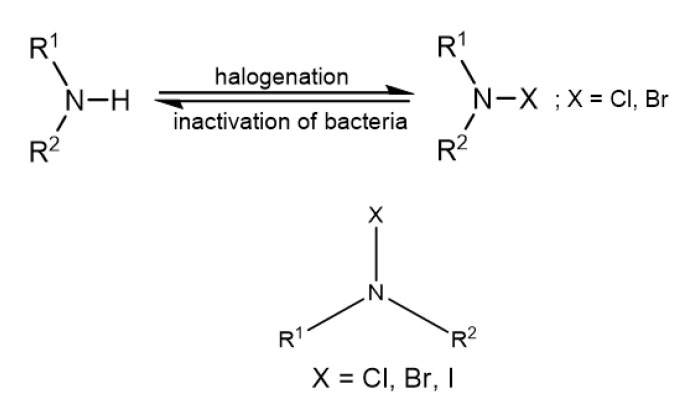
General chemical structure of an *N*-halamine.

*N*-halamines (see Figure 2 for the general chemical structure) have biocidal properties due to the oxidation state of +1 of the halide atom [39].

**Scheme 9:**
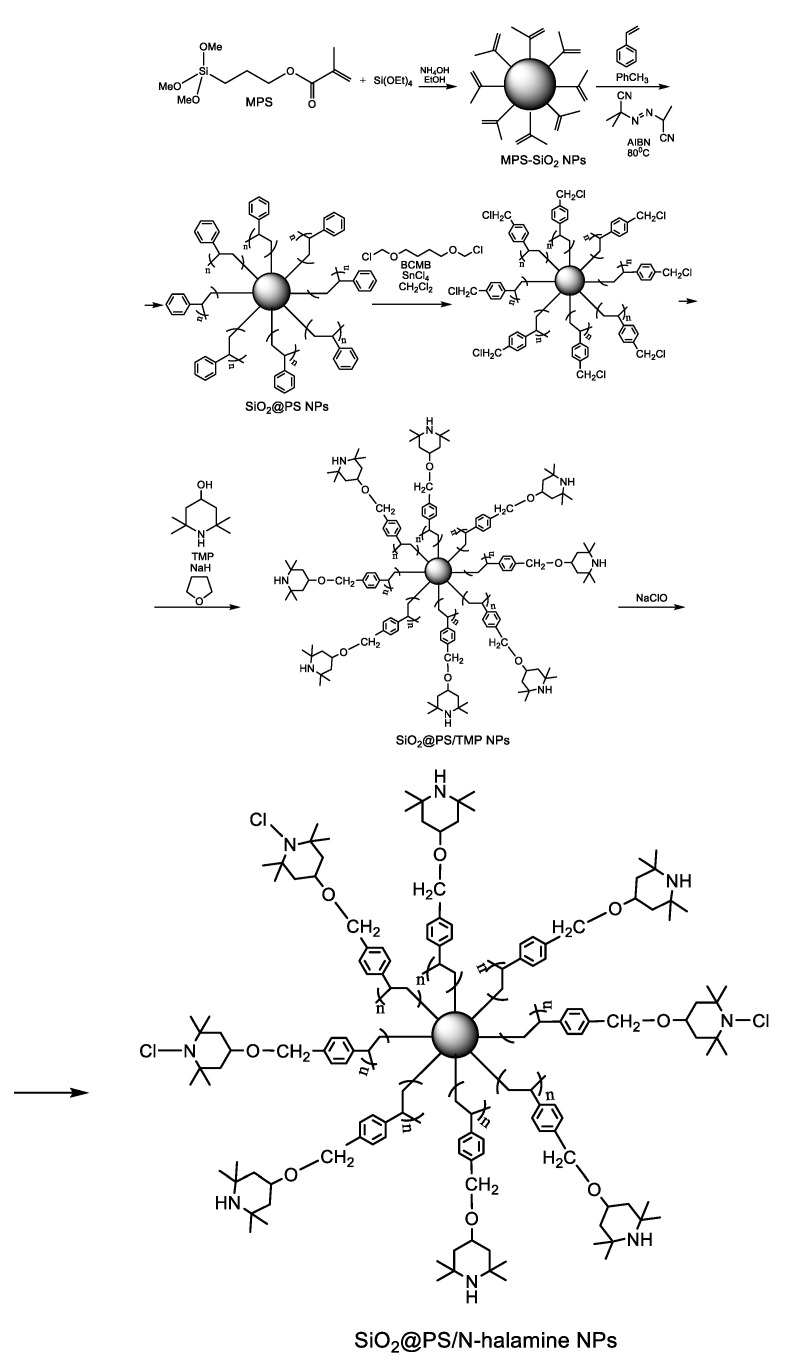
Synthetic pathway to antimicrobial *N*-halamine-functionalized silica nanoparticles [40]. Adapted from an open access source.

Dong et al. [40] prepared novel antibacterial nanoparticles by the immobilization of *N*-halamines onto polystyrene (PS)-coated silica nanoparticles. For the covalent attachment of PS to the surface of the silica nanoparticles, the authors used the “grafting through” methodology. In this synthetic strategy, the surface of the silica nanoparticles is first functionalized with a coupling reagent like 3-(methacryloxy)propyltrimethoxysilane (MPS) in order to introduce polymerizable vinyl groups, which in the presence of a suitable initiator like azobisisobutyronitrile (AIBN) will serve as comonomers in the early stages of further polymerization of styrene [41]. Increasing the MPS grafting density, hybrid silica-PS nanoparticles with well-defined core-shell morphology could be obtained [42]. The higher the MPS grafting density, the higher the probability of oligomeric radicals derived from styrene to be captured by silica, thereby promoting the formation of a PS shell around silica. The synthetic pathway is presented in Scheme 9. In brief, silica nanoparticles were synthesized by the classical method of Stőber through base-catalyzed hydrolysis and condensation of tetraethylorthosilicate (TEOS). Next, MPS was added dropwise to the reaction mixture to afford MPS-modified silica nanoparticles (MPS-SiO_2_ NPs). The silica nanoparticles surface-modified with vinyl groups were mixed with styrene in toluene, and radical polymerization was initiated by adding AIBN. The polymerization reaction was allowed to proceed at 80 °C for 12 h to afford polystyrene coated silica nanoparticles (SiO_2_@PS NPs). Next, the 2,2,6,6-tetramethyl-4-piperidinol (TMP) moiety was covalently immobilized on SiO_2_@PS NPs in two steps. First, the benzene rings of PS were chloromethylated using the noncarcinogenic chloromethylation reagent 1,4-bis(chloromethyoxy)butane (BCMB) in the presence of the Lewis acid catalyst SnCl_4_. Second, the chloromethylated product was treated with TMP in anhydrous tetrahydrofuran (THF) in the presence of NaH to obtain SiO2@PS/TMP NPs. Eventual chlorination was carried out by reacting SiO2@PS/TMP NPs with sodium hypochlorite in a buffered solution at pH 7. The antimicrobial activity of the SiO_2_@PS/*N*-halamine NPs was assessed by the plate counting method using *E*. *coli* as a model bacterium. After incubation, the colonies grown on the plates were counted, and the percent survival was calculated as the ratio between the number of surviving bacterial colonies of the test sample and that of the control *E*. *coli* without antibacterial treatment. No bacterial survival was seen on the culture plate corresponding to the sample that was exposed for 1 h to the SiO_2_@PS/*N*-halamine NPs [40]. As one can expect, the antibacterial activity was dependent on the oxidative chlorine content of the nanoparticles, which was determined by iodometric/thiosulfate titration procedure according to the following equations:

>N-Cl + 2I^−^ + H^+^ → >N-H + I_2_ + Cl^−^ (1)

I_2_ + 2S_2_O_3_^2−^ → 2I^−^ + S_4_O_6_^2−^ (2)

The highest oxidative chlorine content of 2.37% in the SiO2@PS/*N*-halamine NPs was achieved using a concentration of 5 wt % NaClO in the final step and conducting the chlorination reaction at a temperature of 25 °C for 2 h [40]. The unique advantage of *N*-halamines is that they can be repeatedly regenerated after multiple applications by simply reacting them with halogen donor compounds like sodium hypochlorite, sodium hypobromite, trichloroisocyanuric acid, or sodium dichlorocyanurate according to Figure 2 [39,43].

### 5.3. Chlorhexidine

**Figure 3:**
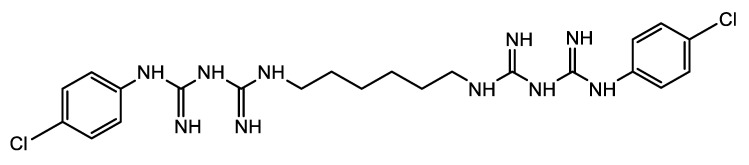
Chemical structure of chlorhexidine.

Chlorhexidine (CHX) (Figure 3) is an antiseptic biguanide compound known for the ability to retain its effectiveness for an extended period [44]. It is widely used as an endodontic irrigant.

Daud et al. [45] immobilized CHX on the surface of medical-grade SS316L stainless-steel disks (SS) through the intermediacy of a pre-deposited PDA film. CHX was subsequently immobilized on the PDA-pre-modified disks by dipping them in CHX digluconate solutions of different concentrations (10, 20, 30, 40, and 50 mM) for 24 h, at room temperature, in the dark. The so-prepared SS-PDA-CHX disks were tested for their cytotoxicity and antibacterial activity. Cytotoxicity was evaluated in vitro on human skin fibroblast cells (HSF 1184, ECACC, UK), both directly and indirectly. In the indirect approach, an MTT assay was used, whereas the direct approach relied on a live/dead viability/cytotoxicity assay. The results showed that at low CHX concentrations, i.e., 10 and 20 mM, neither the viability nor the morphology of HSF cells was significantly altered. The antibacterial activity was assessed both qualitatively using a live/dead bacterial viability kit L13152, and quantitatively by the plate counting method. *E*. *coli* and *S*. *aureus* were used as Gram-negative and Gram-positive, respectively, the bacterium model. The bacterial colonies formed on nutrient agar plates were counted manually and used to calculate the bactericidal ratio (BR) taking into account the CFU determined for the tested sample (SS-PDA-CHX disks), and the CFU determined for the control (SS-PDA disks) according to the following formula, BR = (CFU_control_ − CFU_sample_)/CFU_control_ × 100. BR approached nearly 100% for all SS-PDA-CHX disks.

### 5.4. Usnic Acid

**Figure 4:**
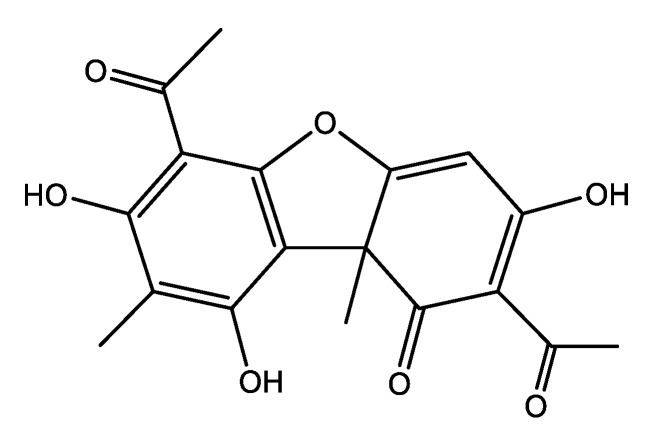
Chemical structure of usnic acid.

Usnic acid (UA) is a secondary metabolite found in lichens with strong antimicrobial activity against *S*. *epidermis*, *S*. *aureus*, *E*. *faecalis*, *Mycobacterium tuberculosis,* and some pathogenic fungi [46]. In spite of these interesting biological properties, the utilization of this dibenzofuran derivative (Figure 4) was limited by its low water solubility and high hepatotoxicity.

The aforesaid properties of UA prompted our group to start a series of research studies aiming to develop novel dosage forms of UA with increased biocompatibility and bioavailability and decreased toxicity.

In a first approach, we developed a novel Fe_3_O_4_ nano-ferrofluid formulation for the magnetically targeted delivery of UA [47]. Briefly, superparamagnetic Fe_3_O_4_ nanoparticles stabilized by an oleic acid (OA) shell were prepared by microwave-assisted synthesis, subjecting a mixture of ferrite nanoparticles and OA to a microwave field for 10 min [48]. Fe_3_O_4_ nanoparticles were prepared by the coprecipitation method following the Massart’s procedure. An extra-shell of the bioactive UA was adsorbed onto the OA-stabilized Fe_3_O_4_ nanoparticles by adding UA into a chloroform dispersion of the prepared Fe_3_O_4_-OA core-shell magnetic nanoparticles and mixing until the complete evaporation of the solvent. The nanoparticles were separated magnetically, air-dried, re-dispersed in chloroform, and the procedure was repeated once more. The antibiofilm activity against *S*. *aureus* was assessed by VCC assay and by CLSM visualization of the *S*. *aureus* biofilm. Both positive (UA-coated coverslips) and negative (coverslips coated with bare Fe_3_O_4_ core-shell nanoparticles) controls were used. Both the qualitative (CLSM) and the quantitative (VCC) assay revealed a great improvement of the antibiofilm activity by incorporation of UA into the nanofluid.

In another work [49], we used the advanced laser deposition technique MAPLE to easily fabricate thin film coatings of polylactic acid (PLA)-polyvinyl alcohol (PVA) microspheres loaded with UA on Ti substrate. The UA-loaded PLA-PVA microspheres were prepared by the emulsion solvent evaporation method. The best films were obtained working with a laser fluence of 300 mJ/cm^2^. The cytotoxicity of the UA-loaded coatings was assessed comparatively with uncoated control substrates using mesenchymal stem cells (MSC) that were stained with fluorescein diacetate (FDA) and subsequently examined by fluorescence microscopy to reveal the potential morphological changes. The outcomes showed that the prepared thin coatings have no adverse effect on the normal morphology and growth of the MSC. VCC assays demonstrated that the newly fabricated UA-loaded coatings inhibit *S*. *aureus* biofilms at all stages from initiation to maturation.

We also succeeded in the MAPLE deposition of thin films consisting of inclusion complexes of UA with γ-cyclodextrin (γ-CD) on double-side polished silicon and glass slides [50]. The optimal compromise between the amount and the structural integrity of the laser transferred material was obtained at the laser fluence of 500 mJ/cm^2^. The biocompatibility of the γ-CD-UA films comparatively with uncoated controls was assessed in vitro on endothelial cells by fluorescence microscopy using the RED CMTPX dye. No cytotoxic effect was observed, a result that was also confirmed by an additional MTT assay. *S*. *aureus* biofilm development on γ-CD-UA-coated silicon and glass materials, and on uncoated controls, respectively, was assessed by the VCC method. The results proved the capacity of the γ-CD-UA coatings to hinder both bacterial attachment and biofilm formation, with the antibiofilm activity being maintained for at least 72 h.

### 5.5. Biofilm Control by Inhibitors or Modulators of Bacterial Signaling Processes

#### 5.5.1. Antibiofilm Coatings with Physically Entrapped or Covalently Immobilized Quorum Sensing Inhibitors

**Figure 5:**
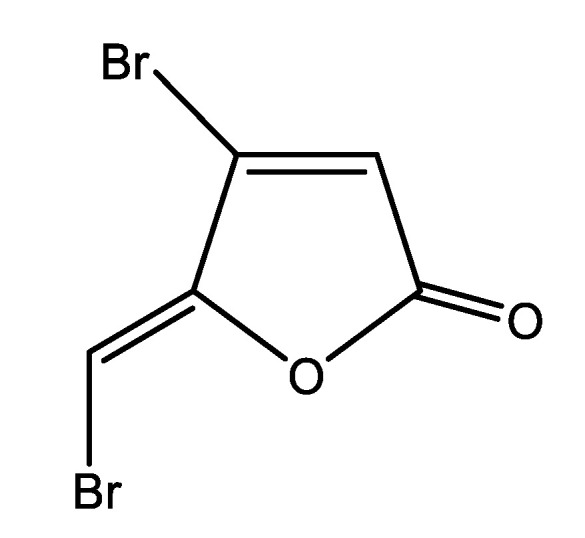
Chemical structure of the quorum sensing inhibitor, (Z-)-4-bromo-5-(bromomethylene)-2(5H)-furanone.

The signal transfer interference approach is another promising strategy to control biofilm infections by interfering with bacterial cell-to-cell communication. By impairing quorum sensing (QS) systems, quorum sensing inhibitors (QSIs) may suppress biofilm formation on medical devices. Wu et al. [51] prepared antibiofilm coatings on glass slides support by incorporating the QSI halogenated furanone compound (Z)-4-bromo-5-(bromomethylene)-2(5H)-furanone (BBF) (Figure 5) into a Nafion polymer matrix.

The antibiofilm activity was tested on a mixed microbial culture and on three pure strains of *P*. *aeruginosa*, *E*. *coli*, and *Bacillus subtilis*. The best results were obtained for a coating prepared by immersing the glass slides support into a 1% Nafion solution that contained 10 mg/L BBF for 12 h. The extent of biofilm formation after various incubation times (6, 12, 24, 48, and 72 h) was indirectly determined using the crystal violet staining assay by measuring the optical density at 600 nm. Compared to uncoated control slides, the glass slides coated with 1% Nafion polymer and 10 mg/mL BBF (BBF-Nafion) showed a biofilm reduction of 51% for the mixed culture and between 23% and 48% depending on the type of the pathogen for the pure strain cultures after 48 h of incubation. The authors also proposed a possible mechanism to explain the synergetic effect on biofilm inhibition observed for the BBF-Nafion coatings compared to the coating with BBF or Nafion alone. They suggested that a large amount of sulfonic acid groups on Nafion are exposed on the outer surface of the composite BBF-Nafion coating, thereby creating a negatively charged surface that repels bacteria, while the active QSI, which is slowly released from the coating, binds to membrane-associated receptor proteins of bacterial cells, thereby impeding their interaction with the native autoinducer signaling molecules and inhibiting the expression of some functional genes.

**Scheme 10:**
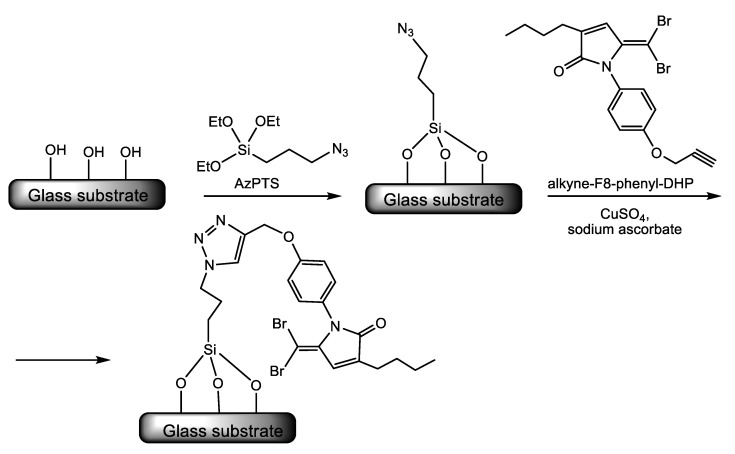
An example is illustrating the click-chemistry strategy used to attach quorum sensing inhibitors dihydropyrrolones (DHPs) to a glass substrate [52]. Adapted with permission from Quorum sensing inhibitory activities of surface-immobilized antibacterial dihydropyrrolones via click chemistry. Copyright (2014) Elsevier.

Ho and co-workers [52] used the extremely versatile click chemistry techniques implying the copper-catalyzed 1,3-dipolar cycloaddition reaction between an azide and an alkyne to form a 1,2,3-triazole ring, which acts as a linker for the attachment of potent QS inhibitors dihydropyrrolones (DHPs) [53] to a glass substrate. Following the procedure exemplified in Scheme 10, the authors synthesized a series of glass substrate attached DHPs and assessed the antibiofilm activity of the functionalized substrates. They proved the ability of surface-attached DHPs to interfere with the QS-controlled transcription of the virulence gene *lasB* in *P*. *aeruginosa* in vitro. *LasB* or pseudolysin is a multifunctional protease that, in the acute stage of *P*. *aeruginosa* infections, degrades the constituents of the host extracellular matrix (ECM), namely elastin, collagen, fibronectin, laminin, and vitronectin provoking tissue injury and hemorrhage. During chronic infections, *lasB* disables or dismantles aspects of the host immune response by degrading or inactivating several components of the immune system [54]. The authors reported a 72% repression of *lasB* gene expression by surface attached DHPs with no affection for cell viability. Moreover, these surface-immobilized QS inhibitors reduced bacterial adhesion up to 97% for both *P*. *aeruginosa* and *S*. *aureus* [52].

#### 5.5.2. Antibiofilm Coatings Are Interfering with c-di-GMP Signaling. Nitric Oxide-Releasing Coatings and Other Small Molecule Modulators of c-di-GMP-Dependent Signal Transduction Pathways

**Scheme 11:**
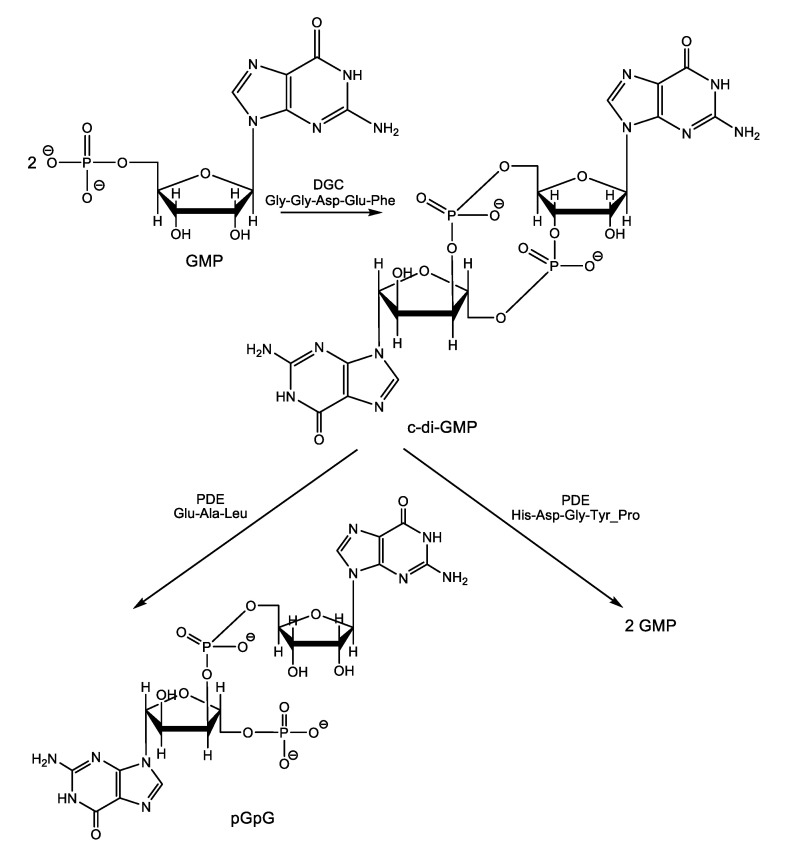
Biosynthesis and biodegradation of c-di-GMP.

The central role played by the second messenger cyclic-diguanosine monophosphate (c-di-GMP, Scheme 11) in biofilm formation and dispersion was well established, although the precise molecular mechanisms of the signal transduction remain mostly unelucidated [55]. However, as we previously pointed out in Part I of this review, it is known that high cellular levels of c-di-GMP promote sessility and biofilm formation by binding to the transcription factor FleQ protein, c-di-GMP down-regulates expression of flagella biosynthesis genes and up-regulates transcription of genes in the pel operon encoding exopolysaccharides (EPS), which are main constituents of the extracellular sheltering matrix required for biofilm formation in *P*. *aeruginosa* [56,57]. Conversely, low c-di-GMP levels down-regulates the production of biofilm matrix components and promote biofilm dispersal and returning to the planktonic mode of life. Targeting c-di-GMP signaling systems appears as a promising way to control biofilms with two major advantages over other therapeutic strategies. First, c-di-GMP-mediated signaling was not observed in higher eukaryotic organisms, and therefore, inhibition of this signal by some small molecules is expected to be harmless or to exhibit low toxicity to the infected host, while strongly affecting bacterial biofilms (selective toxicity). Second, although c-di-GMP regulates the lifestyle of bacteria, it is not necessary for bacterial growth [58] and, therefore, would not select for resistant bacteria as other classical bactericidal and bacteriostatic therapeutic agents usually do.

The intracellular level of this central biofilm life cycle regulator depends on its turn on the rate of c-di-GMP production and degradation. C-di-GMP is synthesized from two GMP molecules by the enzymes called diguanylate cyclases (DGCs) and is degraded under the hydrolytic activity of two types of specific phosphodiesterase enzymes (PDEs), one that linearizes the molecules converting it into 5’-phosphoguanylyl-(3′→5′)-guanosine (pGpG) and another one that split the molecule into two GMP molecules. Both DGCs and PDEs have conserved catalytic domains as follows Gly-Gly-Asp-Glu-Phe for DGCs and Glu-Ala-Leu/His-Asp—Gly-Tyr-Pro for the PDEs producing pGpG and GMP, respectively [59,60].

Nitric oxide (NO) is an endogenous antimicrobial agent produced by macrophages in response to bacterial infection. Nitric oxide is a strong oxidizing agent that damages biological membranes, thereby diffusing inside the cell and altering DNA and proteins.

In 2006, the research group led by Webb reported that the ubiquitous biologically active signal molecule NO is able to induce biofilm dispersal at low, non-toxic concentrations in *P*. *aeruginosa*. Based on their discovery, the authors proposed a novel treatment to eradicate biofilm infections with this pathogen, the combined administration of a NO-donor, and an antimicrobial agent [61]. Later, the same researchers showed that non-cytotoxic NO concentrations stimulate PDEs activity leading to an overall decrease in intracellular c-di-GMP and biofilm dispersal [62]. The precise molecular basis for PDEs activation has been elucidated in *Shewanella woodyi*. Over 250 known bacterial species, including *S. woodyi,* encode heme nitric oxide/oxygen binding (HNOXs) proteins, which act as high affinity NO sensors [63]. When a heme-NO complex forms, the HNOX protein of *S. woodyi* binds and activates a PDE enzyme [64,65].

**Scheme 12:**
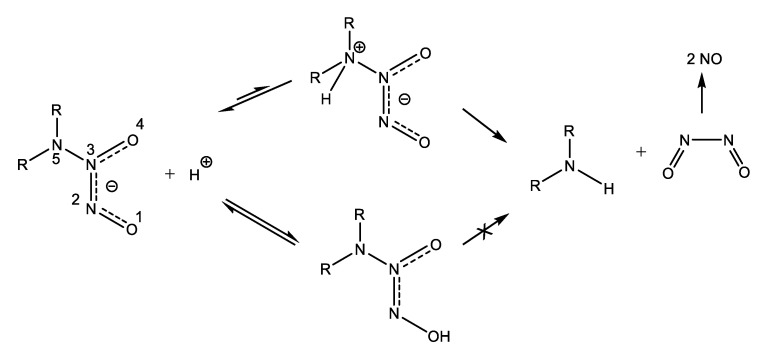
Mechanism of proton-driven NO release from diazeniumdiolates [66]. Adapted with permission from Decomposition of amino diazeniumdiolates (NONOates): Molecular mechanisms. Copyright (2014) Elsevier.

However, the utilization of NO-donor compounds that spontaneously release NO in solution to treat biofilm infections is hampered for two reasons. First, NO is a lipophilic gas that can freely penetrate across cell membranes, and due to the high reactivity imparted by its free radical character, it damages non-selectively both bacterial cells and host cells. Second, the high reactivity and the abundance of available molecular targets cause the half-life time of NO in biological systems to be truly short. Therefore, the successful application of the NO-induced biofilm dispersal approach critically depends on the development of delivery systems capable of targeted on-demand NO release. An important and highly versatile class of NO donors are the diazeniumdiolates (NONOates). These compounds containing the [N(O)NO] functional group are readily available by reacting secondary amines and high pressure of NO. Dialkylamino diazeniumdiolates undergo proton-initiated decomposition leading to secondary amines and NO according to the mechanism depicted in Scheme 12 [66]. Although the oxygen atoms are the preferential sites of protonation, only protonation at the nitrogen atom bearing the two alkyl radicals results in heterolytic N-N bond cleavage leading to eventual NO release.

Barraud et al. [67] developed a new class of targeted NO-donor prodrugs based on cephalosporin-3’-diazeniumdiolates. Due to the clever design of these prodrugs, NO release from a NONOate donor is prevented in the absence of some bacteria-specific enzymes. Thus, bacteria-triggered enzymatic NO release and targeted NO delivery were achieved. 3’-Diazeniumdiolates-substitued cephalosporines spontaneously release the NONOate leaving group following nucleophilic attack from specific bacterial β-lactamases and cleavage of the β-lactamic ring. Moreover, it seems that not only secreted β-lactam antibiotics inactivating β-lactamases can activate the release of NO-donor NONOates since NO release from these prodrugs was also triggered by non-β-lactamase-producing *E. coli* extracts. The authors suggested that other bacterial enzymes, most probably transpeptidases or the transpeptidase domain of penicillin-binding proteins, which are key enzymes involved in bacterial cell wall synthesis at the same time being the target enzymes of β-lactam antibiotics [68], can also activate the release of the NO-donor leaving group from these prodrugs. The innovative 3’-diazeniumdiolates-substitued cephalosporines were tested on several pathogens, including mixed species biofilms from cystic fibrosis (CF) sputum, and they proved to be highly effective in biofilm dispersal. Moreover, they can potentiate the antimicrobial activity of therapeutic agents currently used in the treatment of chronic lung diseases, including chronic obstructive pulmonary disease (COPD) and reduce biofilm tolerance to antibiotics. For instance, when co-administered with tobramycin or ciprofloxacin, they significantly decrease the number of colony-forming units (CFU) in *P*. *aeruginosa* biofilms and also enhance the susceptibility to azithromycin of *Haemophilus influenzae* biofilms [69].

**Scheme 13:**
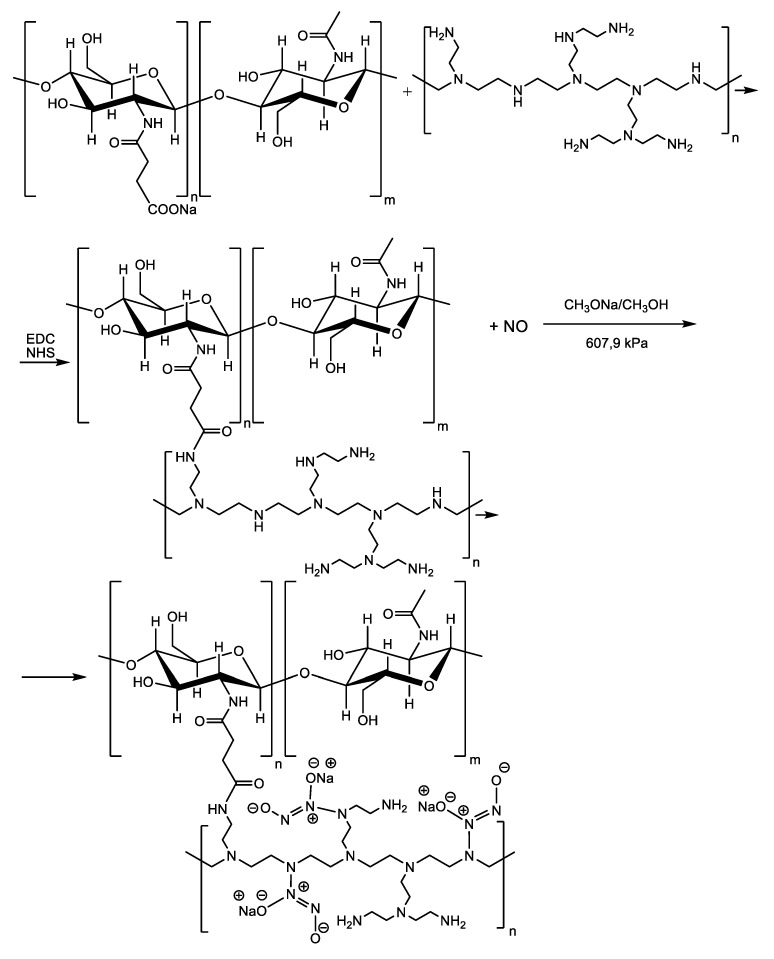
Synthesis of *N*-succinyl chitosan sodium (CPCS) cross-linked to branched poly(ethyleneimine (PEI) NONOate NO donor conjugates.

Besides acting as a signal that triggers biofilm dispersal, NO can be used to actively inhibit bacterial proliferation or kill bacteria when it is administered in a controlled manner and in the appropriate concentration to avoid cytotoxic effects on host cells. So, there are some challenges to be overcome for the safe and effective application of NO donors in clinical practice. First, it is a problem of NO donor stability under physiological conditions and second the ability to constantly deliver NO amounts ranging in the therapeutic window for a prolonged period of time. Keeping this in mind, Ji et al. [70] used branched PEI scaffold with plenty of secondary amino groups to cross-link *N*-succinyl chitosan sodium (CPCS) through amide linkage as depicted in Scheme 13. The intermediate CPCS cross-linked to branched PEI are gel materials capable of sustained release and were further treated with NO at a gas pressure of 70 psi for seven days to yield NONOate conjugated polymeric materials with a considerable increase in NO donor groups per mass unit. An overall amount of 2.031 μmol NO was released in 5 h per mg material prepared at a molar ratio between CPCS and branched PEI of 1:4. The CPCS-branched PEI-NONOate conjugates showed excellent inhibition of both *S*. *aureus* and *E*. *coli* as well as low toxicity towards healthy mammalian fibroblasts.

**Figure 6:**
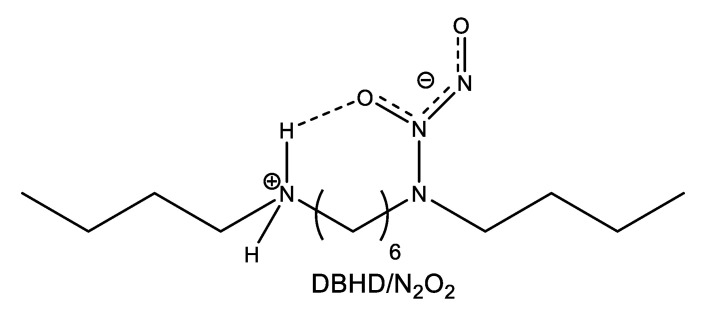
The chemical structure of dibutylhexyldiamine diazeniumdiolate.

The group of Meyerhoff [71] incorporated the lipophilic dibutylhexyldiamine diazeniumdiolate (DBHD/N_2_O_2_) (Figure 6) into the hydrophobic, biodegradable, and biocompatible polymer PLGA. To minimize diazeniumdiolate or amine product leaching, the polymer matrix was further coated with a protective top coating of silicone rubber (SR). Since decomposition of the diazeniumdiolate NO donor is a proton-driven reaction, the pH within the inner polymer matrix can modulate the kinetics of NO release from these coatings. From this point of view, the matrix chosen by the authors is especially suited for controlled release applications as polyester PLGA can provide protons through its intrinsic carboxyl end groups and through its hydrolysis products, i.e., lactic acid (LA) and glycolic acid (GA). Hence, nicely exploiting the above property, the authors were able to prepare coatings with tunable NO release kinetics by controlling the rate of PLGA hydrolysis, which in turn is adjustable with the LA/GA ratio in the PLGA polymer. First, the authors used two types of PLGA, both having the same LA/GA ratio (50/50) but differing by the nature of the end groups: Methyl ester terminated PLGA that contained no acid groups (capped polymer) and -COOH terminated PLGA (uncapped polymer). The uncapped 50/50 PLGA showed a much higher NO starting flux compared to the capped material due to the presence of the terminal carboxyl groups that donate protons to promote DBHD/N_2_O_2_ decomposition and NO release. Moreover, the water uptake of the bilayer coating caused the hydrolysis of the PLGA layer. While NO was released, the produced DBH diamine remained in the film-forming a quaternary ammonium hydroxide, which in turn may act as a basic catalyst accelerating the rate of PLGA hydrolysis. Consequently, the NO flux decreased rapidly with elapsed time due to the fast depletion of the NO donor. The hydrolysis rate could be slowed down by increasing the LA ratio in the PLGA polymer. Using a capped 75/25 PLGA, the NO release period could be extended up to eight days, while methyl ester terminated poly(lactic acid) (capped poly(lactic acid)) released NO for 10 days at 37 °C. The antibiofilm activity of the prepared NO-releasing coatings was evaluated under dynamic conditions. Various PLGA/SR coatings containing 30% weight DBHD/N_2_O_2_ and differing from one another by the LA/GA ratio and controls were incubated in a flow bioreactor at 37 °C with Gram-positive *S*. *aureus* or Gram-negative *E*. *coli* cultures for seven days. Two types of controls were used in these experiments: An uncapped 50/50 PLGA/SR bilayer and a SR layer alone. On the eighth day, the films were taken out of the reactor, stained with bacterial live/dead dyes, and biofilm growth was visualized with a fluorescence microscope. Compared to the control SR surface, the capped poly(lactic acid)/SR bilayer coating with 30% weight DBHD/N_2_O_2_ produced about 98.4% reduction in biofilm biomass of *S*. *aureus* and about 99.9% reduction for *E*. *coli* [71].

Recently, Sadrearhami et al. [72] took advantage of the unique adhesive ability of PDA to nicely use a PDA-coated glass substrate as a versatile scaffold for subsequent surface functionalization by grafting of low-fouling polymer (PEGylation) and introduction of *N*-diazeniumdiolate moieties as NO precursors. PDA is rich in quinoide and indoline species (Scheme 6). The grafting of the hydrophilic low-fouling polymer is achieved through Michael addition of quinone functionalities with amino or thiol groups or by Schiff base formation, while the secondary amine indoline groups form *N*-diazeniumdiolates on purging with NO gas. The NO release rate is tunable by controlling the thickness of the PDA film. The antibiofilm activity of the NO releasing coatings was tested using Gram-negative *P*. *aeruginosa* (i.e., wild-type PAO1 and multidrug-resistant PA37) and Gram-positive *S*. *aureus* (ATCC 29213). Biofilm development on *N*-diazeniumdiolates functionalized substrates was inhibited by 96 and 70% after exposure to bacterial culture solution for 24 and 36 h, respectively. The authors ascribe the excellent outcome to synergic combination of the antifouling properties imparted by pegylation and antibacterial activity of NO. The killing efficiencies of this coatings against surface attached PAO1, PA37, and *S. aureus* bacteria were, 99.9, 97, and 99%, respectively.

The other enzymes involved in controlling of c-di-GMP intracellular levels, namely DGCs, also represent a potentially promising target for antibiofilm drugs to be exploited. Based on an in silico pharmacophore screen study, Sambanthamoorthy et al. [73] were the first scientists to identify four low molecular mass lead compounds acting as inhibitors of DGC enzymes. All four DGC inhibitors (LP 3134, LP 3145, LP 1062, LP 4010) prevented biofilm formation by *P*. *aeruginosa* and *A*. *baumannii* in a continuous-flow system and dispersed *P*. *aeruginosa* preformed biofilms. Only LP 3134 was able to disperse preformed *A*. *baumannii* biofilms. The authors also conducted studies to determine the impact of the four lead compounds onto the first step in biofilm development, namely the primary adhesion of bacteria to a surface. They found out that only LP 3134 effectively inhibits *P*. *aeruginosa* adherence to a surface, while none of the compounds could impede *A*. *baumannii* adherence. Growing *P*. *aeruginosa* as a biofilm on 14-French urethral catheters in the presence and absence of LP 3134 and LP 3145, the authors proved the ability of both compounds to reduce biofilm formation on catheters surface. When cytotoxicity to eukaryotic cells was assessed, only LP 3134 and LP 4010 showed to lack of toxicity on human keratinocytes. Based on all the above results, the authors draw the conclusion that LP 3134 is the most promising as a drug candidate DSCs inhibitor.

#### 5.5.3. Biofilm Disruption by Targeting the Stringent Response Alarmone (p)ppGpp

**Figure 7:**
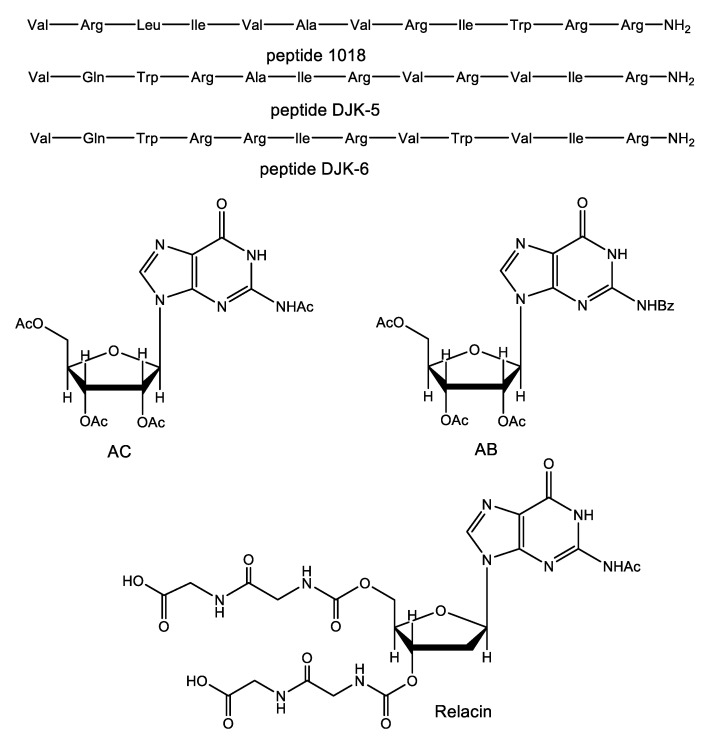
Inhibitors of the synthesis of the (p)ppGpp alarmone.

(p)ppGpp is a second messenger essential for virulence and long-term persistence of pathogenic bacterial biofilms. Three AMPs, namely 1018, DJK-5, and DJK-6 (see Figure 7 for the amino acids sequences) were shown to inhibit the synthesis and trigger degradation of (p)ppGpp in both Gram-positive and Gram-negative bacteria resulting in a reduction of biofilm development and increased susceptibility towards AMPs [74,75]. Pletzer et al. [76] demonstrated that treatment with peptides DJK-5 and 1018 suppressed the activity of the spoT promoter in a *P*. *aeruginosa* murine cutaneous infection model causing reduction of the size of the skin lesions and decreasing the bacterial burden inside the abscess. Wang, de la Fuente-Núñez et al. [77] proved that the application of a combined treatment using peptide 1018 and the classical oral disinfectant chlorhexidine on hydroxyapatite greatly reduces the volume of the dental plaque consisting in highly antibiotic-resistant multispecies biofilms. Similarly, the peptide DJK-5 alone or mixed with EDTA increases the antibiofilm activity of root canal irrigants such as sodium hypochlorite in endodontic therapy [78].

Syal et al. reported significant inhibition of the synthetase activity of the bifunctional Rel enzyme by the acetylated (AC) and, respectively, acetylated benzoylated (AB) guanosine derivatives shown in Figure 7. Rel protein is responsible for the synthesis and degradation of the (p)ppGpp alarmone in mycobacteria. The authors used a *Mycobacterium smegmatis* strain cultured in the presence of AC and AB compounds for in vivo assessment of the effect of these compounds on (p)ppGpp synthesis. They found a sharp drop in the (p)ppGpp levels corroborated with significant disruption of biofilm formation [79]. The two guanosine synthetic analogs also inhibited biofilm formation by the pathogen *Mycobacterium tuberculosis*. Toxicity tests, namely MTT assay with H460 cells and hemolysis assay on healthy red blood cells (RBCs), showed compounds AC and AB as being nontoxic. In comparison to relacin, a previously developed (p)ppGpp production inhibitor [80,81], AC and AB showed improved biofilm disruption performances. To the best of our knowledge, no coating formulations have been developed yet for any of the substances in the present paragraph.

### 5.6. Silver Nanoparticles and Silver Ions

Antimicrobial properties of silver have been well-known for a long time, but the mechanism underlying silver nanoparticles-mediated toxicity is still incompletely understood, and the full details of how silver nanoparticles induce toxicity in bacterial cells remain to be elucidated. However, a series of studies have provided important mechanistic insights into the antibacterial properties of both zero-valent silver nanoparticles and silver ions [82,83,84,85].

#### 5.6.1. Cell Membrane Damage

Two main mechanisms have been proposed for the silver nanomaterials-induced cell membrane damage. According to the hard and soft Lewis acids and bases theory, silver, a soft acid, has a high tendency to form strong covalent bonds with soft bases such as phosphorus and sulfur compounds. Since the bacterial cell membrane is rich in sulfur-containing proteins, these ones are likely to be the preferential sites for silver nanoparticles binding. Denaturation of cell membrane proteins affects membrane integrity resulting in increased permeability and eventual disruption of the cell membrane [86]. Sondi and Salopek-Sondi [87] envisaged another mechanism for the cell membrane damage by silver nanoparticles. Using SEM and transmission electron microscopy (TEM) these authors revealed the formation of irregular-shaped pits in the outer membrane of *E*. *coli* cells treated with nanosized silver particles as well as accumulation of the silver nanoparticles in the bacterial membrane and significant increase in membrane permeability due to progressive disruption of the tight packing of lipopolysaccharide (LPS) molecules in the cell wall. A third mechanism relies on the electrostatic attraction between positively charged silver nanoparticles and negatively charged cell membranes, but such a mechanism fails to explain the adhesion and uptake of negatively charged silver nanoparticles. In a recent paper, Radzig et al. [88] reported the strong antibiofilm activity of silver nanoparticles capped by hydrolyzed casein peptides (8.3 nm in diameter) against *E*. *coli* AB1157, *P*. *aeruginosa* PAO1, and *Serratia proteamaculans* 94. Studying the effect of several factors such as mutations in genes involved in DNA repair, mutations in porin genes, as well as LuxI/LuxR QS systems on the bacterial sensitivity towards silver nanoparticles, the authors concluded that the OmpF and OmpC porins-mediated transport of silver ions into the cell is mainly responsible for the antibacterial activity of silver nanoparticles. This study brings direct evidence of the major effect bacterial cell wall penetration by silver nanoparticles has on bacterial cell viability. However, this mechanism of toxicity requires partial (surface) oxidation of silver nanoparticles followed by the release of the chemisorbed Ag^+^ ions formed on the particle’s surface [89]. Release of Ag^+^ ions from silver nanoparticles is induced by molecular oxygen [90,91] according to Equations (3) and (4) below:

4Ag + O_2_ → 2Ag_2_O (3)

2Ag_2_O + 4H^+^ → 4 Ag^+^ + 2H_2_O (4)

#### 5.6.2. Dissipation of the Proton Motive Force

The ability of submicromolar concentrations of silver ions to induce the collapse of the electrochemical proton gradient across the inner membrane of the bacterial cell envelope due to the massive proton leakage occurring through the Ag^+^-modified membrane has been proved by Dibrov et al. and Lok et al. [92,93]. Hence, Ag^+^ ions have a protonophore- and an uncoupler of oxidative phosphorylation-like effect. Dissipating the proton-motive force, Ag^+^ ions cause a depletion of intracellular ATP levels. Translocation of newly synthesized precursors of envelope proteins across the inner membrane is powered by ATP hydrolysis. In turn, ATP is synthesized by oxidative phosphorylation using the energy stored in the form of the proton electrochemical gradient established across the inner membrane. Consequently, in the absence of the proton motive force and ATP, the precursor proteins are not translocated and accumulate in the cytoplasm. The accumulation of the envelope protein precursors in the cytoplasm of *E*. *coli* cells treated with silver nanoparticles was interpreted by Lok et al. as indicating dissipation of proton motive force and depletion of intracellular levels of ATP [93].

#### 5.6.3. Impairment of the Respiratory Chain and Enhancement of ROS Production

Silver nanoparticles interact with thiol groups from protein complexes of the electron transport chain (ETC) causing the inefficient passage of electrons at the terminal oxidase and, hence, incomplete reduction of molecular oxygen and increased production of ROS (superoxide anion radical, hydrogen peroxide, and hydroxyl radicals) [94,95,96,97,98,99,100]. ROS are known to induce oxidative stress in the cells by attacking all vital biomolecules: lipids, proteins, and DNA, thereby provoking breakdown of the plasma membrane, inactivation of key enzymes, and DNA damage. However, ROS are produced as a natural side-product of the respiratory chain, but their damaging action is controlled by a series of enzymes such as the superoxide radical scavenging enzyme superoxide dismutase, catalase, and peroxidase. Silver ions-induced inactivation of these enzymes corroborated with excessive ROS production due to impaired respiratory chain function produces an imbalance resulting in oxidative stress and cell death. Moreover, silver nanoparticles themselves are able to produce highly reactive hydroxyl radicals in a Fenton-like reaction, as depicted by Equation (5) [100].

Ag + H_2_O_2_ −> Ag^+^ + HO^-^ + HO• (5)

#### 5.6.4. Denaturation of Bacterial Proteins and DNA Molecules

Besides their capacity to form stable bonds with thiol group-containing proteins, silver ions might also catalyze the formation of disulfide bridges between cysteine residues, thereby altering proteins conformation and deactivating important enzymes [101]. Using Fourier transform infrared spectroscopy (FTIR), Arakawa et al. [102] showed that silver ions form two types of complexes with DNA by binding to guanine N7 and adenine N7 (type I complex) and to guanine-cytosine and adenine-thymine base pairs (type II complex) at a higher cation/polynucleotide ratio. The morphological changes that occurred in *E*. *coli* and *S*. *aureus* bacterial cells following exposure to silver ions have been directly observed by Feng et al. [103] through TEM imaging and X-ray microanalysis. The TEM micrographs of pristine cells revealed a remarkable electron-light region in the center of the cells, very rich in phosphorus as shown by energy-dispersive X-ray spectroscopy, which comprises the bacterial DNA. After treatment with silver ions, the electron-light region collapsed to form an electron-light dot indicating that DNA condensation took place. Similar results were obtained by Li et al. [104]. The authors assumed that this behavior was a self-defense mechanism; bacteria are conglomerating their DNA to protect it against attack from silver nanoparticles. However, this impeded DNA replication since DNA replication can be effectively conducted only when DNA molecules are in a relaxed state and not in a condensed one [105].

Despite the excellent antimicrobial properties of silver nanoparticles [106], care must be taken in their use since silver nanoparticles appear to have toxic effects in higher organisms, including humans as well. The more complex structural and functional organization of eukaryotic cells can be affected by silver nanoparticles through various mechanisms such as induction of programmed cell death via activation of the p53-mediated signaling pathway [107].

To end this discussion of the antibacterial mechanisms of silver, meaning both zero-valent silver nanoparticles and silver ions, we feel that the most appropriate conclusion is that one drawn by Kedziora et al. [108]. Based on previous comparative studies like the one of Li et al. [109], the opinion of these authors is that once one accepts the presumption that silver nanoparticles act as silver ions sources, the suggestion that silver ions and silver nanoparticles have similar action mode appears as a reasonable one. However, because of scarce knowledge of mechanisms underlying the antibacterial activity of zero-valent silver nanoparticles and of how the particular synthetic method impacts these mechanisms, the possibility of major differences between silver nanoparticles and silver ions in the molecular basis of action and bacterial resistance cannot be ruled out.

The synthetic routes to prepare silver nanoparticles can be classified as: (i) Physical methods, (ii) chemical methods, and (iii) biological methods [110,111]. The most commonly used chemical method is based on chemical reduction and involves a metal precursor, a reducing agent, and a stabilizing/capping agent that prevent nanosilver from aggregation in solutions or on surfaces.

Vijayakumar et al. [112] choose an environment-friendly green synthesis [113] to prepare alginate stabilized silver nanoparticles (SA-AgNPs). The synthesis was carried out in the water as an environmentally benign solvent, using silver nitrate as a metal precursor, sodium borohydride as a reducing agent, and the biopolymer sodium alginate as a capping agent. Briefly, a solution of sodium alginate in deionized water was prepared, and solutions of the metal precursor and of the reducing agent were successively added to it under magnetic stirring. The reaction mixture was eventually dialyzed against water to remove any unreacted AgNO_3_, NaBH_4_, and salts formed during synthesis. The SA-AgNPs were freeze-dried [112]. The ecotoxicity of prepared SA-AgNPs was assessed on *Ceriodaphnia cornuta*, a freshwater crustacean, in comparison with sodium alginate and silver nitrate. The SA-AgNPs were considerably less toxic than AgNO_3_. Silver nitrate caused 100% mortality of *C*. *cornuta* neonates at a concentration of 10 μg/L, while a four-fold increase in concentration was necessary to attain the same mortality when SA-AgNPs were used in place of AgNO_3_. The estimated LC_50_ values were 7.94 and 3.2 mg L^–1^ for SA-AgNPs and AgNO_3_, respectively [105]. Although being less toxic than AgNO_3_, SA-AgNPs still induced physiological abnormalities (reduced swimming, heart rate, and thoracic limb movement) in *C*. *cornuta*. The antibiofilm activity of SA-AgNPs was tested against Gram positive (*Listeria monocytogenes*) and Gram negative (*Vibrio parahaemolyticus*) bacteria. The authors reported biofilm inhibition of 98% for *L*. *monocytogenes* and of 84% for *V*. *parahaemolyticus* at 75 μg/L SA-AgNPs concentration [112].

Khan et al. [114] succeeded in their attempt to achieve silver nanoparticles biosynthesis by using the functional groups of biomolecules present in a *Heliotropium crispum* (HC) plant extract as both reducing and stabilizing/capping agents. In brief, HC-AgNPs have been prepared by simply mixing the plant extract at a certain concentration with a solution of AgNO_3_ in deionized water for 3 h at 37 °C. The antibiofilm properties of HC-AgNPs were evaluated on clinical isolates of MRSA, *P*. *aeruginosa*, and *A*. *baumannii* using SEM imaging. All tested strains were found to be susceptible to HC-AgNPs. As compared to untreated controls, the SEM micrographs of bacterial strains samples incubated with silver nanocomposites treated media clearly showed biofilm disruption, changes in the morphology of bacteria, and destruction of the extracellular polymeric substances (EPS) that play an important role in cell aggregation, cell adhesion, and biofilm formation [114].

Santhosh and Kandasamy [115] prepared new hybrid inorganic-organic nanocomposite coatings by incorporating silver ions doped TiO_2_ into a polymeric epoxy matrix. This material has the advantage of combining the magnificent biocidal properties of silver ions and the photocatalytic antibacterial activity of nano-TiO_2_ (anatase) [116]. The nanocrystalline anatase TiO_2_ was prepared by the acid-catalyzed hydrolysis of titanium butoxide. The doping agent (AgNO_3_) was dissolved in aqueous acetic acid, and the resulting solution was added dropwise to an ethanolic solution of titanium butoxide. The so-prepared Ag-TiO_2_ nanocomposite contained about 1.5% wt. of Ag^+^. Incorporation of Ag-TiO_2_ into the epoxy resin polymer matrix was achieved in three steps as follows: (i) Preparation of the resin mixture containing the commercially available resin Lapox^®^ L-12 (liquid epoxy resin based on bisphenol-A) and the reactive diluent, Lapox^®^ XR-19 (poly(propylene glycol) diglycidyl ether), (ii) decrease the viscosity of the resin mixture by diluting it with ethanol at 1:5 ratio, and (iii) addition of corresponding amounts of the previously prepared Ag-TiO_2_ nanocomposite followed by sonication eventually yielding epoxy polymeric nanocomposites with 0.5, 1.0, 1.5, and 2.0 wt% Ag-TiO_2_ loading [115]. The antibiofilm activity was indirectly evaluated through the crystal violet staining assay by the measurement of crystal violet absorbance at 600 nm, using a destaining solution. The results were given as inhibition percentages of biofilm development of the tested bacteria (*E*. *coli* and *S*. *aureus*). The antibiofilm activity was directly proportional to Ag-TiO_2_ loading. Under UV irradiation for 24 h, the polymer nanocomposite containing 1.5 wt% Ag-TiO_2_ completely inhibited (100%) biofilm formation of both *E*. *coli* and *S*. *aureus* [115].

Table 1 summarizes the active antibiofilm strategies and the corresponding nanocoatings dealt with throughout the Section 2, Section 3, Section 4 and Section 5.

## 6. Combining Active and Passive Antibiofilm Strategies

Sileika et al. [117] designed new surface coatings with dual functionality that are able to release a bacteria-killing agent and to repel the bacterial adhesion simultaneously. The synthetic procedure starts with a quite simple “priming” method through which an adhesive PDA film was deposited on a polycarbonate substrate by a dip-coating process. The method takes advantage of the chemical structural features of the PDA film. The catechol groups in PDA are redox-active and can get oxidized to quinone groups, thereby reducing metal ions like Ag^+^ [118]. On the other hand, quinone functional groups can serve as anchor points for covalent grafting through Michael addition of hydrophilic polymers bearing nucleophilic end groups like poly(ethylene glycol) methyl ether thiol (mPEG-SH). Briefly, the successive synthetic steps followed by the authors were the next ones: (i) Coating PDA thin films on the polycarbonate substrates through direct immersion of the substrates in a freshly prepared solution of DA hydrochloride in bicine buffer (pH 8.5) for 18 h at room temperature; (ii) formation and incorporation of zero-valent silver nanoparticles into the PDA coatings by simply incubating the PDA-modified substrates with a solution of silver nitrate for 24 h at room temperature; (iii) deposition of a new PDA layer by repeating the first step under identical conditions except for the incubation time which was reduced to 4 h; and (iv) grafting PEG brushes by incubation of the silver nanoparticles incorporated PDA coatings in a solution of mPEG-SH in cloud point buffer for 6 h at 55 °C [117]. The prepared coatings exhibited sustained release of silver nanoparticles over at least 10 days. The performances of these coatings in terms of their antifouling and antimicrobial properties were evaluated on three bacterial strains: *E*. *coli*, *S*. *epidermidis*, and *P*. *aeruginosa*. The bacterial attachment was assessed using fluorescence imaging, while a live/dead staining assay was used to estimate the antibacterial activity. While unmodified polycarbonate substrates developed biofilms of all three bacterial strains, substrates modified with PDA, silver nanoparticles, and PEG brushes suppressed bacterial attachment for all the tested strains. When the PDA/Ag/PEG-modified polycarbonate substrates were inoculated with an inoculum suspension (20 μL; 2.5 × 10^9^ CFU/mL) of each bacterial strain premixed with a live/dead stain and incubated at 37 °C for 24 h, almost all bacterial cells were found to be dead at the end of the incubation period except for *E*. *coli* for which a slightly lower ratio of the area occupied by dead cells to the total area covered by bacterial cells (both live and dead) was observed [117].

**Scheme 14:**
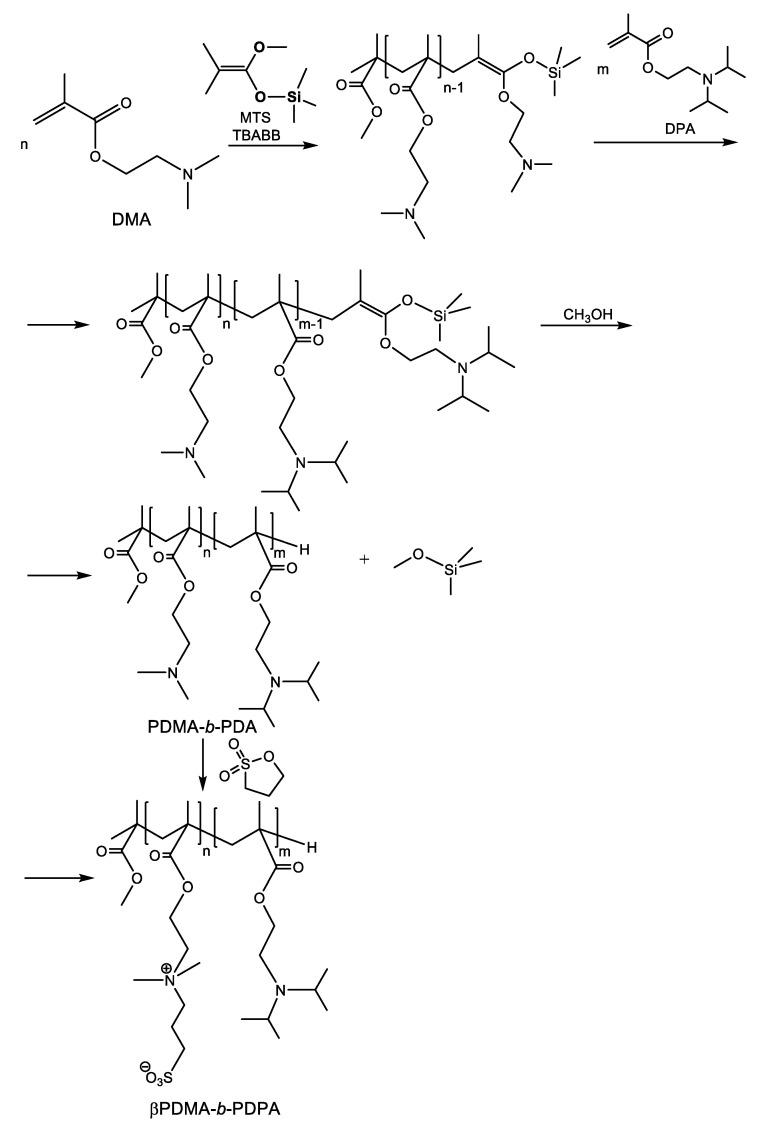
Synthetic pathway for the preparation of zwitterionic βPDMA-*b*-PDPA diblock copolymer micelles, the building blocks for further construction of mono and multilayered films.

Erel-Goktepe and coworkers reported an ingenious and innovative approach to creating smart dual-functional surface coatings with both anti-adhesive and antibacterial agent releasing properties. They prepared monolayered films [119] as well as LbL self-assembled multilayer coatings [120] based on zwitterionic block copolymer micelles. Using a diblock copolymer composed of a polyzwitterionic poly[3-dimethyl (methacryloyloxyethyl) ammonium propane sulfonate] block (βPDMA) imparting anti-biofouling properties and a pH-responsive poly[2-(diisopropylamino)ethyl methacrylate] (PDPA) block, the authors attained pH-induced micellization by increasing the pH of an aqueous acidic solution of the βDMA-*b*-PDPA diblock copolymer above the pK_a_ value of about 6.4 of the PDPA block. By increasing pH, the PDPA block becomes uncharged due to gradual deprotonation. Hence, the hydrophobic association among the PDPA blocks is reinforced, and the βPDMA-*b*-PDPA diblock copolymer self-assembles in micellar aggregates with hydrophilic zwitterionic bacteria repelling β-PDMA coronae and hydrophobic PDPA cores, the latter serving as reservoirs to trap hydrophobic antimicrobial agents. The diblock copolymer was prepared by group transfer polymerization (GTP). GTP is a controlled living polymerization technique that allows polymerization of acrylate monomers in the presence of a silyl ketene acetal as an initiator and nucleophilic or electrophilic catalysts [121]. More specifically, the synthesis started with polymerization of the first monomer that is 2-(dimethylamino)ethyl methacrylate (DMA) initiated by 1-methoxy-1-trimethylsiloxy-2-methyl-1-propene (MTS) and conducted in the presence of tetra-*n*-butyl ammonium dibenzoate (TBABB) as a nucleophilic oxyanion catalyst [122] under rigorously anhydrous conditions in an inert nitrogen atmosphere. Polymerization of the resulted living PDMA homopolymer was continued further with the second monomer that is 2-(diisopropylamino)ethyl methacrylate (DPA) and eventually the living diblock copolymer was quenched by adding methanol. Selective betainization of the DMA residues in the PDMA-*b*-PDPA diblock copolymer was achieved by treatment with 10 mol % excess (based on DMA residues) of 1,3-propane sultone at room temperature. Scheme 14 depicts the whole synthetic pathway used to prepare the βPDMA-*b*-PDPA diblock copolymer [119,120,123]. The diblock copolymer was dissolved in 1 mM NaH_2_PO_4_ buffer at pH 3.0 to achieve a final concentration of 0.1–0.2 mg/mL. Micellization occurred upon the gradual addition of a 0.1 M NaOH solution up to pH 7.5. Self-assembled monolayers of βPDMA-*b*-PDPA micelles were prepared using a simple dip-coating technique by immersing the silicon wafer or glass slides substrates into an aqueous solution of the diblock copolymer at pH 7.5 for 3 h. The deposition process was driven electrostatically through attractions between the quaternized amino groups of the zwitterionic β-PDMA-coronal chains and the partially negatively charged silanol groups of the substrate. To evaluate the adhesion of *S*. *aureus* on substrates modified with these zwitterionic micellar coatings, the authors performed a series of complementary tests including viable cell counting, microscopic fluorescence assays using live/dead staining, and phase-contrast imaging as well as crystal violet staining assay. The outcomes of these tests revealed the capacity of βPDMA-*b*- PDPA micellar monolayers to reduce biofilm formation by 45% and short-term adhesion of bacterial cells by up to 95% as compared to control unmodified substrates. Moreover, the authors prepared β-PDMA-*b*-PDPA micelles loaded with the hydrophobic antimicrobial agent triclosan [120]. First, triclosan was dissolved in ethanol at a concentration of 2.5 mg/mL and 1 mL of this solution was diluted 200-fold in a 1 mM NaH_2_PO_4_ buffer at pH 3.0. Next, the β-PDMA-*b*-PDPA diblock copolymer was introduced in the above solution, and micellization was induced by raising the pH to 7.5. Multilayer coatings were fabricated by LbL assembly of βPDMA-*b*-PDPA micelles and poly(sodium 4-styrene sulfonate) (PSS) at pH 7.5. The build-up of triclosan containing multilayered films was achieved through alternate immersion of silicon wafers or glass slides into solutions of triclosan-loaded βPDMA-*b*-PDPA micelles and PSS with rinsing after each immersion step. The first deposited layer was always the zwitterionic copolymer micelles layer. The number of layers in the multilayered coatings was found to be of crucial importance for the anti-adhesive properties against *S*. *aureus*, the best results at physiological pH being obtained for a three-layer film configuration [120]. Three-layer films also displayed significant anti-adhesive properties at pH 7.5 against *E*. *coli*. Furthermore, pH-triggered smart triclosan released from the multilayer films was achieved by dropping the environmental pH to 5.5. pH lowering results in gradual protonation of the amine groups in the hydrophobic PDPA core, which renders them hydrophilic, thereby causing disassembly of the micellar aggregates and release of the encapsulated triclosan. Since at infections sites, the surrounding milieu is slightly acidic, the authors concluded that besides their bacterial anti-adhesive properties, the pH-induced release of antibacterial agents is another important advantage of these mono and multilayered coatings [120].

**Scheme 15:**
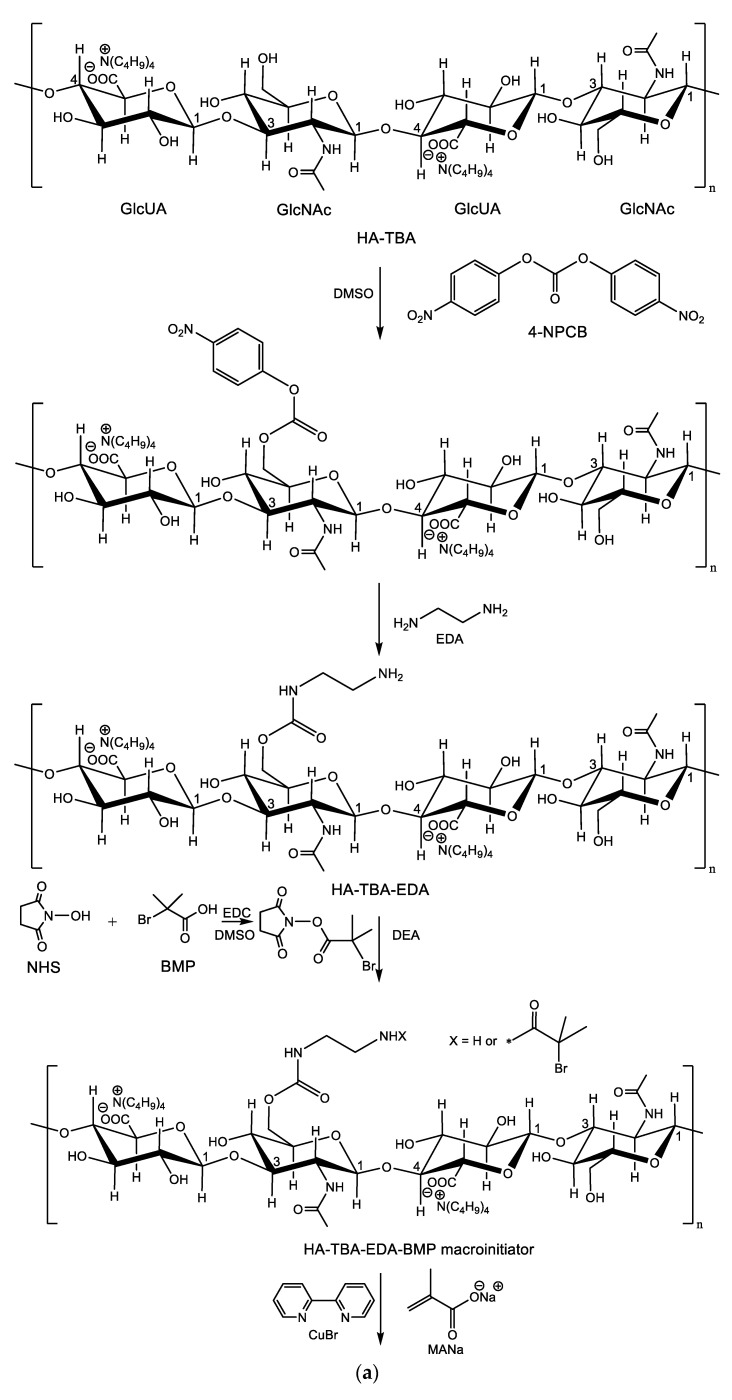

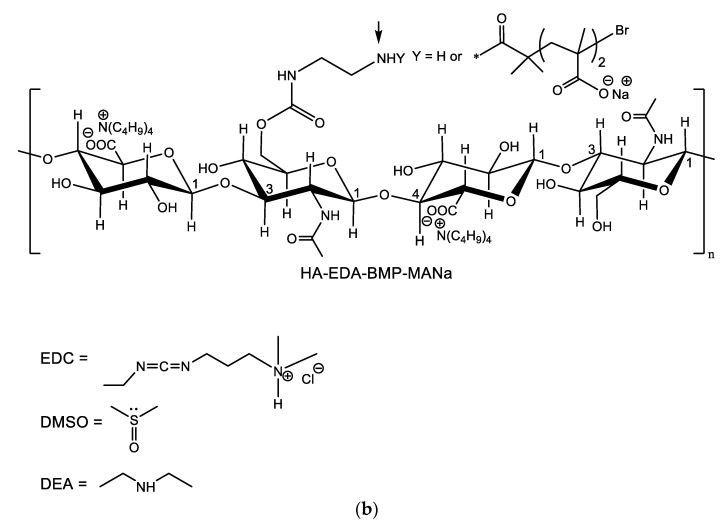
(**a**) Synthetic pathway for the preparation of hydrogels with non-fouling and controlled drug release properties based on a graft copolymer derivative of HA. (**b**) Synthetic pathway for the preparation of hydrogels with non-fouling and controlled drug release properties based on a graft copolymer derivative of HA.

Palumbo et al. [124] prepared new hydrogels with non-fouling and controlled drug release properties based on a graft copolymer derivative of HA. HA is a linear polyanionic polysaccharide composed of repeating units of *D*-glucuronic acid (GlcUA) and *D*-2-*N*-acetyl-glucosamine (GlcNAc), which are linked together by β-1,4, and β-1,3 glycosidic bonds. The graft copolymer was prepared by ATRP. The synthesis depicted in Scheme 15 starts with the activation of some of the primary alcohol functionalities in the tetrabutylammonium salt of HA (HA-TBA) as *p*-nitrophenyl carbonate derivatives followed by displacement of the very good leaving group *p*-nitrophenoxy by ethylenediamine (EDA). The grafted amino groups served as nucleophilic counterparts for reaction with the activated *N*-hydroxysuccinimidyl (NHS) ester of 2-bromo-2-methylpropionic acid (BMP) to eventually yield the ATRP macroinitiator, HA-TBA-EDA-BMP [125,126]. The next step was the graft polymerization of sodium methacrylate (MANa), which occurred from the modified HA backbone in the presence of copper bromide Cu(I)Br as a catalyst and of 2,2’-bipyridyl (Bpy) as a ligand to afford the graft copolymer HA-TBA-EDA-BMP-MANa. The newly synthesized HA derivative bore both pendant unreacted ethylenediamine groups and carboxylic moieties from the newly grafted short segments of polymethacrylate. When dispersed in aqueous buffers at various pH values (3.0, 6.0, and 7.4) and concentrations ranging from 5 to 10% (*w*/*v*), the polycarboxylic/amino-functionalized derivative of HA formed transparent hydrogels, which were used to enclose the antibiotic vancomycin. Depending on the initial pH of the dispersing medium, the corresponding final pH values of the obtained hydrogels were 5.0, 6.0, and 7.0 at a polymer concentration of 10% *w*/*v*. For the preparation of vancomycin-loaded hydrogels, the antibiotic was first dissolved in each of the above three dispersing media, and then appropriate volumes of each of the resulted solutions were mixed with lyophilized copolymer powders in the right ratios to produce final formulations containing 2% *w*/*v* vancomycin and 10% *w*/*v* copolymer. In vitro release studies were performed on Franz diffusion cells for all the three medicated hydrogels prepared from the graft copolymer derivative of HA. The cumulative amount of vancomycin released during a period of time by each hydrogel as compared to the total amount of antibiotics released over the same period of time from a vancomycin formulation prepared similarly except that unmodified HA was used.

All the three hydrogels obtained from the HA-TBA-EDA-BMP-MANa graft copolymer showed a much-diminished burst effect as compared to the sample prepared using HA. The lower the final pH of the hydrogel, the more important the effect on burst release reduction. For instance, the hydrogel with the final pH equal to 5.0 released about 24% of vancomycin after 5 h, while 45% of the drug was released during the same time interval from the hydrogel formulated at pH 7.0. For comparison, after 5 h, the formulation obtained using unmodified HA released 85% of the embedded vancomycin. Moreover, after the initial burst effect, all three hydrogel formulations based on the HA-TBA-EDA-BMP-MANa graft copolymer showed sustained release profile, the hydrogel with the lowest pH value being the most effective in slowing down drug release rate and exhibiting extended-release up to 48 h [124]. The authors ascribed this behavior to the pH-induced physical cross-linking occurring at lower pH values as a consequence of the aggregation of the methacrylic acid portions. Protonation of the carboxylate moieties in the short polymethacrylate segments reinforced the hydrophobic interactions of the α-methyl groups. On the contrary, increasing the pH values, more and more carboxylic moieties became deprotonated, and the system appeared less compact due to electrostatic repulsions between the negatively charged carboxylate groups [124,125]. An antimicrobial efficacy test was performed using a hydrogel batch with the following characteristics: a final pH value equal to 5.0, a polymer concentration of 10% (*w*/*v*) and an initial vancomycin concentration equal to 50-fold the MIC that is 50 μg/mL or 1% *w*/*v*. The test showed that the amount of vancomycin released by this hydrogel was sufficient to inhibit *S*. *aureus* growth for 24 h. This is an important result since it suggests that the hydrogel formulation could effectively control the pathogen’s growth making external administration of vancomycin no longer necessary [124]. Furthermore, to evaluate comparatively the efficacy of the above hydrogel against *S*. *aureus* biofilm formation, the authors used hydroxyapatite doped titanium discs coated with various antibacterial coatings: HA, vancomycin-loaded HA, HA-TBA-EDA-BMP-MANa hydrogel, vancomycin-loaded HA-TBA-EDA-BMP-MANa hydrogel with a final pH of 5.0, as well as uncoated control discs. After *S*. *aureus* inoculation, adhesion and formation of bacterial colonies were assessed using SEM imaging. Uniformly distributed clusters of *S*. *aureus* were observed on the surface of uncoated titanium discs. The degree of colonization was decreased significantly for HA-coated discs and even more for the vancomycin-loaded HA coatings. No bacterial adhesion could be detected for the substrates coated with the hydrogels based on the HA-TBA-EDA-BMP-MANa copolymer, no matter whether the hydrogel was unloaded or loaded with vancomycin. The authors concluded that due to the synergistic combination of the anti-biofouling properties and drug release modulating capabilities, their newly developed hydrogels based on a graft copolymer derivative of HA bearing pH-responsive functionalities could represent a biomatrix suitable for the coating of implantable orthopedic prostheses.

**Figure 8:**
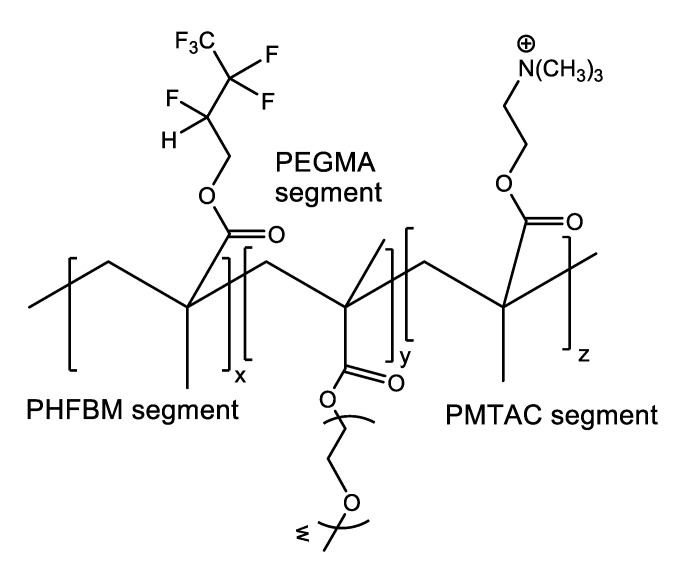
Amphiphilic block-like copolymer membrane surface modifier for the creation of an integrated active-passive fouling release nanocoating.

Gao et al. [127] modified the surface of polyvinylidene difluoride (PVDF) membranes fabricated through the non-solvent induced phase separation method with an amphiphilic block-like copolymer nanocoating consisting of three polymeric segments with distinct properties, specifically, low surface energy poly(hexafluorobutyl methacrylate) (PHFBM), hydrophilic poly(poly(ethylene glycol) methyl ether methacrylate) (PEGMA), and antibacterial poly[2-(methacryloyoxy)ethyl trimethylammonium chloride] (PMTAC) (Figure 8). The amphiphilic membrane surface modifier was obtained via AIBN initiated free radical polymerization. In the course of PVDF membrane fabrication, during the phase inversion process, the hydrophilic PEGMA and PMTAC segments spontaneously segregate at the water/membrane interface, while the hydrophobic PHFBM segment undergoes forced surface segregation being dragged by the spontaneously segregated hydrophilic segments to which is covalently linked. Thereby, the PVDF membrane is endowed with both passive antifouling and active contact killing properties. Integration of the active and passive antifouling mechanism resulted in reaching nearly 100% efficiency against *E*. *coli* and *S*. *aureus* surface colonization.

## 7. Applying Active and Passive Strategies One at a Time and Reversibly Switching between the Two Approaches

Another successful antibiofilm strategy based on combining into a single platform, both non-fouling and bactericidal properties, consists of developing a surface that is able to execute both functions sequentially, rather than simultaneously as presented in the previous paragraph. The aim is to design smart surfaces that in response to some environmental changes are able to irreversibly switch from an antimicrobial status to a bacterial repelling status (the so-named “kill and repel” strategy) or reversely from a non-fouling status to a bacterial killing status (the so-called “resist and kill” strategy) [128].

In the “kill and repel” approach, the surface is initially in the bactericidal status and kills bacteria on contact. Subsequently, the surface switches to the non-fouling status releasing the attached bacteria and preventing any further bacterial association with the surface. As an illustrative example, we mention here the pioneering work of Cheng et al. [129] who were the first to report a switchable polymer surface coating based on the contact killing polymer poly(carboxybetaine methacrylate ethyl ester) (PCBMAEE) which after pH-triggered irreversible hydrolysis turns into the non-fouling zwitterionic polymer poly(carboxybetaine methacrylate) (PCBMA). PCBMAEE killed more than 99.9% of *E*. *coli* K12 in 1 h, and 98% of the dead bacterial cells could be released after its hydrolytic conversion to PCBMA [129].

**Scheme 16:**
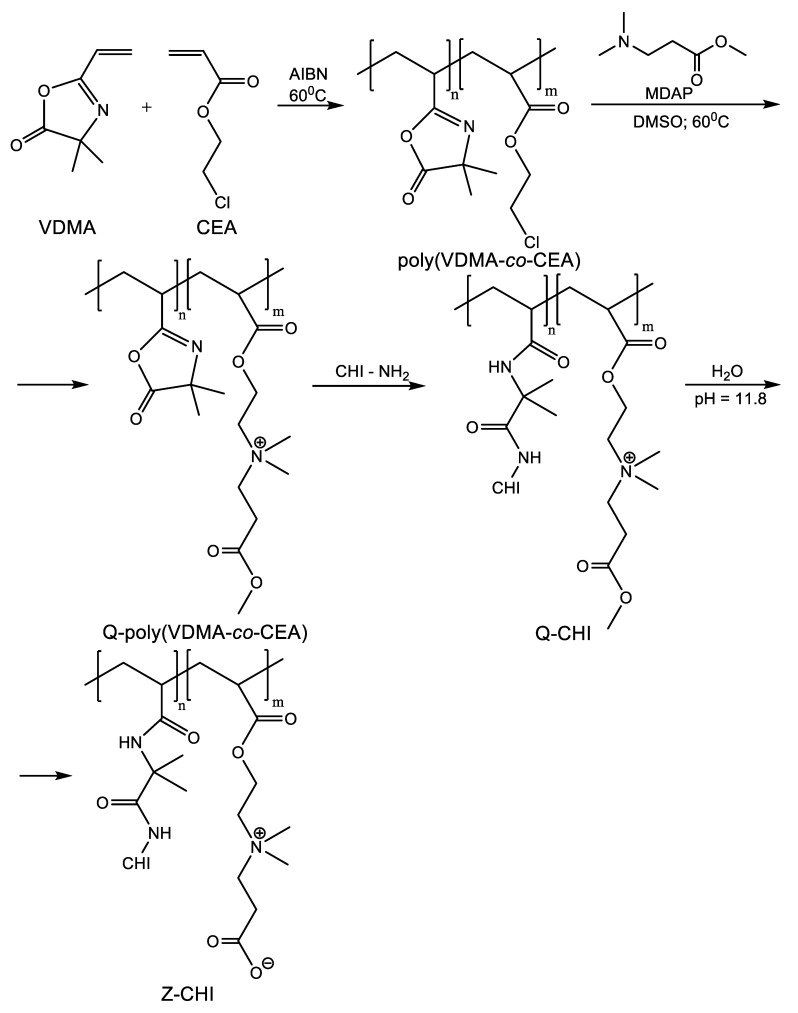
Synthesis of a bactericidal quaternized chitosan (CHI) non-woven substrate surface and its conversion to the non-fouling zwitterionic form [130]. Adapted with permission from Facile fabrication of bactericidal and antifouling switchable chitosan wound dressing through a ‘click’-type interfacial reaction. Copyright (2015) Elsevier.

Wang et al. [130] conceived a wound dressing based on a modified CHI non-woven surface which can be kept in a bactericidal status during storage and undergoes a hydrolytic-driven switching to its non-fouling status before being used. A presynthesized copolymer bearing azlactone moieties and positively charged quaternary ammonium groups was attached to the CHI backbone through a “grafting to” methodology based on highly efficient “click” type ring-opening of pendant azlactones by the attack of the nucleophilic primary amino groups in CHI. In brief, copolymerization of 2-vinyl-4,4-dimethylazlactone (VDMA) with 2-chloroethyl acrylate (CEA) was conducted in the presence of AIBN as an initiator, and subsequently, methyl 3-(dimethylamino)propionate (MDAP) was *N*-alkylated with the pendant 2-chloroethyl acrylate groups in the resulting copolymer to afford the quaternized Q-poly(VDMA-*co*-CEA) copolymer as shown in Scheme 16. Next, dried CHI non-woven was dipped into dimethylsulfoxide (DMSO) solution of the quaternized copolymer for 24 h at 60 °C, rinsed with DMSO and ethanol, and dried at room temperature [130]. The antibacterial performance of the as-prepared modified CHI non-woven surface was estimated by counting the number of adhered *E*. *coli* bacterial cells revealed by SEM imaging. The surface density of attached *E*. *coli* on the quaternized CHI (Q-CHI) non-woven substrate was drastically reduced compared to a CHI non-woven reference demonstrating its strong antibacterial effect. Conversion of the bactericidal Q-CHI non-woven surface into the non-fouling zwitterionic CHI (Z-CHI) non-woven surface was achieved simply by basic hydrolysis in a solution containing 1.0 M NaOH and methanol with a pH value equal to 11.8. The Z-CHI non-woven surface-displayed very low hemolytic activity, reflecting low cytotoxicity. A hemolytic ratio as low as 0.44% was determined using blood freshly drawn from healthy rabbits. Furthermore, the reduced hemolytic activity translates into a good antithrombotic effect since hemolysis has been associated with increased platelet aggregation and accelerated clot formation. The blood clotting index of the Z-CHI non-woven surface determined after 1 h of incubation at 37 °C was larger than 95%, indicating good antithrombosis properties [130].

Temperature is another environmental stimulus that can be used to trigger the transition of a smart, functional coating from its bactericidal status to the non-adhesive status. To this purpose, Laloyaux et al. [131] fabricated smart thermoresponsive copolymer brushes based on diethylene glycol methyl ether methacrylate (MEO_2_MA), hydroxyl-terminated oligo(ethylene glycol) methacrylate (HOEGMA), and 2-hydroxyethyl methacrylate (HEMA) by SIATRP. The copolymer brushes were grown from plasma oxidized silicon wafers modified with the ATRP initiator, 2-bromo-2-methyl-propionic acid 3-trichlorosilanyl-propyl ester following the procedure of Brown et al. [132]. The obtained poly[MEO_2_MA-*co*-HOEGMA-*co*-HEMA] copolymer was further conjugated to an antimicrobial peptide derived from natural magainin I by introducing an additional cysteine residue at the C-terminus. Bioconjugation was achieved via a two-step process by using *N*-(*p*-maleimidophenyl)isocyanate (PMPI) as a heterolinker [133]. In the first step, PMPI-grafted brushes were obtained through the reaction of the isocyanate moiety in the heterolinker with the free pendant hydroxyl groups in the HOEGMA and HEMA segments. Subsequently, the reactive maleimide units in the PMPI-grafted copolymer brushes were utilized to conjugate the thiol group of the modified antimicrobial peptide MAG-Cys. At lower temperatures, the copolymer brush is in a swollen extended-chain conformation due to the hydration of the oligo(ethylene glycol) segments. Hence, in this extended-chain conformation, the copolymer brush exposes its grafted antimicrobial magainin I derivative molecules to bacteria enabling interaction with the bacterial cell membrane and inducing bacterial lysis. As the temperature is raised, dehydration occurs, and the copolymer brush collapses into a more compact structure. However, the transition from the extended-chain conformation to the collapsed conformation is not a sharp one but rather a gradual cooperative transition that occurs over a large range of temperatures and not sharply at one temperature. Collapse first occurs at the bottom of the swollen brush, and phase separation propagates upward to the top of the brush during the ongoing process. Hence, the outermost chain segments of the oligo(ethylene glycol) methacrylate-based brush are dehydrated later and collapse at a higher temperature than the inner bulk segments of the copolymer brush. The collapse transition temperature of the bulk of the poly[MEO_2_MA-*co*-HOEGMA-*co*-HEMA] copolymer brushes, $T_{coll}^{bulk}$, grafted from silanized silicon wafer substrates, was determined by Laloyaux et al. [134] through quartz crystal microbalance with dissipation monitoring (QCM-D) measurements in water. $T_{coll}^{bulk}$ can be finely tuned by varying the monomer composition of the polymerization mixture. For instance, increasing the molar composition of the copolymer brush in the more hydrophilic HOEGMA segments results in an increased $T_{coll}^{bulk}$ as compared to the $T_{coll}^{bulk}$ of a poly(McEO_2_MA) brush which equals 22.2 °C. By contrary, increasing the content of the copolymer brush in HEMA segments results in a decrease of the $T_{coll}^{bulk}$. In fact, this is the great advantage this type of copolymer brushes has, since $T_{coll}^{bulk}$ can be adjusted to a value very close to the physiological temperature [135]. A relevant example is a poly[MEO_2_MA-*co*-HOEGMA-*co*-HEMA] copolymer brush with a segment composition of 50:30:20 whose $T_{coll}^{bulk}$ is equal to 35 ± 1 °C. On the other hand, the authors also determined the collapse transition temperature of the surface of the brush, $T_{coll}^{surf}$, by measuring the equilibrium water contact angles, and found out that $T_{coll}^{surf}$ is about 8 °C higher than $T_{coll}^{bulk}$ [131,134]. That means that at a temperature slightly higher than $T_{coll}^{bulk}$, for instance, at 38 °C, the outermost oligo(ethylene glycol) segments still remain in a swollen hydrated state providing bacterial repellent properties. Yet, the amphipathic antimicrobial peptide molecules attached to these chains are no longer exposed but rather tend to hide slightly deeper in the more hydrophobic environment provided by the collapsing brush. The paper of Laloyaux et al. is the first one reporting on a surface that switches from cell-killing to cell-repellent upon increasing temperature and a promise for further development of medical device coatings exhibiting antibacterial properties at room temperature, which after their implantation, turn into non-fouling coatings at the body temperature.

The “resist and kill” strategy was used by Wang et al. [136] to impede bacterial adhesion to medical devices during the first 24 h after implantation, a period that is viewed as being decisive for biofilm formation. Their approach is based on the build-up of a multilayer film by a LbL self-assembly process. The multilayer film was deposited on silicon wafers or glass slides substrates previously cleaned by the Piranha solution and covered with a precursor layer of the branched cationic polyelectrolyte PEI. In fact, the film was composed of two distinct coatings stacked one upon another. The first deposited coating consisted of ten heparin (HEP)/CHI bilayers in which the two polysaccharides self-assembled by electrostatic attraction between the negatively charged HEP and the positively charged CHI. This coating has a CHI-ending layer conferring bacterial contact killing properties. The second coating comprised 10 polyvinylpyrrolidone (PVP)/poly(acrylic acid) (PAA) bilayers. Unlike the construction of the first coating, the build-up of the second coating was based on a hydrogen bond-driven self-assembly process. Hydrogen-bonding LbL coatings are pH-sensitive and prone to disassembly at neutral or basic pH as the carboxylic acid moieties undergo deprotonation resulting in breakage of the hydrogen bonds. Hence, the upper (PVP/PAA)_10_ multilayer coating constructed at pH 4.0 was readily disassembled in a few minutes in PBS buffer at pH 7.4 at 37 °C. Therefore, to resist bacterial adhesion for 24 h, the disassembly rate of the upper coating had to be slowed down, which was achieved by thermal treatment at 110 °C for 16 h when covalent cross-linking of PAA segments through anhydride groups occurred. Since the anhydride moieties are readily hydrolyzable, the disassembly rate of the upper coating could be controlled by the cross-linking density. It is this continuous removal of the outermost surface that impedes bacteria adhesion. After complete top-down destruction of the upper (PVP/PAA)_10_ coating, the bottom bactericidal (HEP/CHI)_10_ coating was eventually exposed. The authors demonstrated the enhanced antibiofilm properties of these coatings against *S*. *aureus* used as a model bacterium. Only a few viable bacterial cells could be observed by SEM and fluorescence microscopy imaging on the surface of (HEP/CHI)_10_-(PVP/PAA)_10_ multilayer film coating samples that were cross-linked as previously described [136].

Surfaces that irreversibly change their status from initial bactericidal to non-fouling can be categorized as “one-time-switch” surfaces since they cannot be restored to their initial form. The already mentioned basic hydrolysis of cationic polycarboxybetaine esters to zwitterionic polycarboxybetaines (Scheme 16) is a convenient way to irreversibly modify surface status. However, to resist bacterial adhesion and biofilm formation for a prolonged period of time, the development of smart materials capable of repeatedly switching between the active contact killing and the passive non-fouling status is highly desirable. Obviously, that cannot be achieved unless the two forms of the surface can be reversibly transformed into each other. Three different approaches have been employed so far to gain the reversibility of the switching process.

One approach makes use of a reversible cyclization/ring-opening reaction. Cao et al. [137,138,139] developed reversibly switchable surfaces based on a series of zwitterionic poly(hydroxy carboxybetaines) (pCB-OH) that can undergo reversible ring closure to the corresponding cationic six-membered ring lactone forms (pCB-Ring). The 2-((2-hydroxy-3-(methacryloyloxy)propyl)dimethylammonio)acetate monomer CB-OH 1 was grown via SIATRP from a gold-coated surface modified with the previously synthesized ATRP initiator mercaptoundecyl bromoisobutyrate to yield the polymer brush pCB-OH 1. The acid-catalyzed lactonization equilibrium CB−OH 1⇄CB−Ring 1 was almost completely driven towards the lactone side by using a strong acid catalyst like trifluoroacetic acid (TFA). The cationic bactericidal pCB-Ring 1 surface was obtained by dipping pCB-OH surface in TFA for 20 min., followed by repeated washing with acetonitrile. Under dry conditions, the pCB-Ring 1 polymer brush surface killed more than 99.9% of the *E*. *coli* K12 bacteria cells sprayed on it in less than one hour [137]. Wetting with neutral or basic aqueous solutions produced hydrolysis of the lactone rings, converting the pCB-Ring 1 surface to the zwitterionic cell-repellent pCB-OH 1 surface. The latter released 90% of the attached dead cells and also prevented further bacterial colonization [137]. The pCB-Ring 1 surface could be regenerated from the pCB-OH 1 surface in an acidic non-aqueous environment. To conclude, the smart polymer material developed by these authors might be used to coat implantable medical devices. The pCB-Ring 1 surface coating kills the pathogenic bacteria attached to the medical device under dry conditions, while after implantation, upon contact with body fluids, the coating switches to the non-fouling zwitterionic pCB-OH 1 form, thereby resisting biofouling. Another potential application of this material is hospital environmental sanitation; bacteria can be killed by the cationic pCB-Ring 1 form of the polymer brush, the dead bacteria released by the pCB-OH 1 form, and, since the surface can be regenerated to its initial bactericidal form, the kill-release cycle can be repeated for several times without significant loss of the initial bactericidal activity. Similar results were obtained by B. Cao et al. [140] who additionally introduced a hydrogen bond-forming hydroxyl group in the CB-OH 2 monomer in order to enhance the mechanical strength of the coatings.

**Scheme 17:**
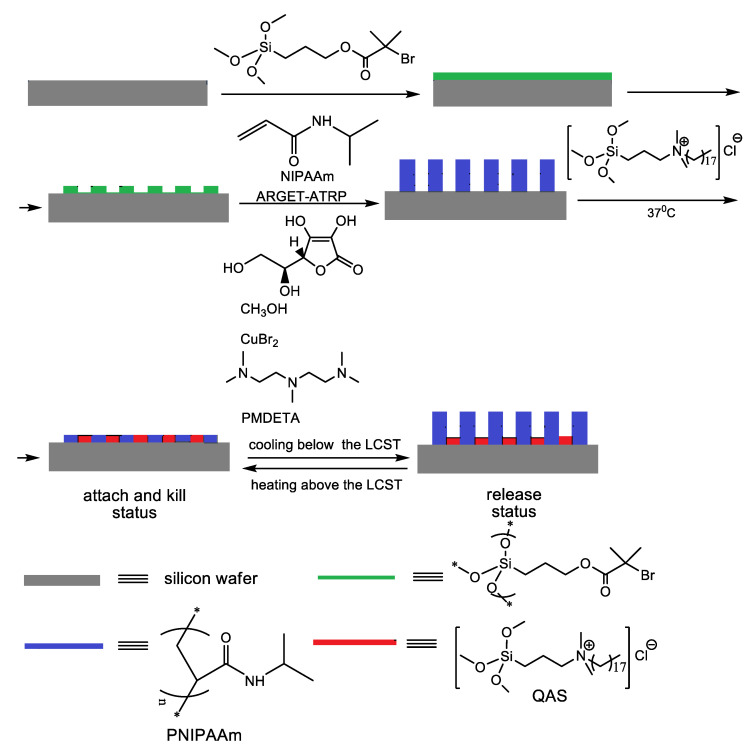
Synthesis of repeatedly switchable bioactive interfaces based on smart temperature-responsive polymer brushes [142]. Adapted with permission from Nanopatterned smart polymer surfaces for controlled attachment, killing, and release of bacteria. Copyright (2013) American Chemical Society.

A completely different switching mechanism was developed by Lopez and co-workers [141,142]. In their approach, the switching between the bacteria attach and kill status and the bacteria release status does not involve any chemical modification of the surface as in the preceding example, but is rather based on reversible and repeated changes in the hydration state of a thermoresponsive polymer brush. Using a combination of interferometric lithography (IL) [143] and surface-initiated activator regenerated by electron transfer atom transfer radical polymerization (ARGET-ATRP) [144], the group of Lopez built up a smart surface comprising a nanopatterned thermoresponsive polymer brush/biocidal quaternary ammonium salt composite. First, a self-assembled monolayer (SAM) of the ATRP initiator (3-trimethoxysilyl)propyl 2-bromo-2-methylpropionate was deposited on the freshly cleaned silicon wafer substrate. Next, the SAM of the ATRP initiator was spatially patterned at the nanoscopic scale by means of UV-IL. Using a two-beam interference system (the so-called Lloyd setup), the authors created a sinusoidally shaped concentration gradient of the ATRP initiator at the silicon wafer surface. At the destructive interference zones where dark fringes are formed, the ATRP initiator stays unaffected by the UV light beam, whereas at the constructive interference zones where bright fringes are formed, the high intensity of the UV beam decomposes the ATRP initiator as schematically illustrated in Scheme 17 [143]. Consequently, using the IL nanopatterning technique, one gains a very precise spatial control of polymer brushes growth, endowing them with dual functionality. The periodicity of the patterns is easily tunable by varying the interference angle. Thermoresponsive poly(*N*-isopropylacrylamide) (PNIPAAm) polymer brushes were grown from the aforesaid nanopatterned surfaces via surface-initiated ARGET-ATRP polymerization of NIPAAm using 1,1,4,7,7-pentamethyldiethylenetriamine (PMDETA) as a ligand, CuBr_2_ as an oxidatively stable Cu(II) species, and ascorbic acid as a reducing agent. The troughs between the nanopatterned parallel PNIPAAm lines have been subsequently filled with the biocidal quaternary ammonium salt (QAS), [3-(trimethoxysilyl)propyl]ammonium chloride. To this end, the nanopatterned PNIPAAm surfaces were incubated for 2 h in 1% aqueous QAS solution at 37 °C, a temperature that is higher than the lower critical solution temperature (LCST) of PNIPAAm. At this temperature, the polymer chains are hydrophobic and collapse, allowing the access of bacteria to the QAS layer covering the inter-spacing between the nanopatterned PNIPAAm lines. This state renders the nanopatterned PNIPAAm/QAS composite surface sticky and bactericidal. Hence, bacteria adhere to the surface being killed by the QAS. Lowering the temperature below the LCST, PNIPAAm becomes hydrated and swollen with an extended chain conformation, thereby hiding the biocidal QAS. In this state, the surface releases the attached dead bacteria, and a being cell repellent, prevents further bacterial adhesion. Increasing the temperature again above LCST, the initial bactericidal status of the nanopatterned hybrid surface can be restored. The authors tested the biocidal activity and bacterial release ability of these PNIPAAm/QAS composite surfaces against *E*. *coli* K12 using QAS and nanopatterned PNIPAAm surfaces as controls. The sample surfaces were inoculated with a bacterial inoculum (1 × 10^8^ CFU/mL) and incubated at 37 °C for 2 h, and the viability of the attached cells was evaluated by a standard dead/live staining assay. When examined by fluorescence microscopy, most cells attached to the QAS control surface showed red fluorescence due to uptake of the membrane-impermeable propidium iodide dye, indicating that the cell envelope was badly damaged resulting in loss of the cell integrity and cell’s death. In contrast, most cells attached to the patterned PNIPAAm surfaces were viable cells as indicated by the green fluorescence of the SYTO 9 dye, which means that these surfaces exhibit no intrinsic bactericidal activity. On the other hand, the nanopatterned PNIPAAm/QAS hybrid surfaces displayed bactericidal activity, although as compared to QAS surfaces, this activity was reduced from 90 ± 2% to 73 ± 5%. However, cooling down the samples to 25 °C (below the LCST of PNIPAAm), the bactericidal activity of nanopatterned PNIPAAm/QAS composite surfaces drastically decreased [142]. The authors explained this behavior through the temperature-triggered reversible switch between the dehydrated collapsed state of the PNIPAAm polymer brush, which exposes the biocidal QAS moieties and the hydrated extended conformation of the PNIPAAm chains that hides the bactericidal compound. Regarding the capability to release the attached cells, this was poor for the control QAS surface. In contrast, 81 ± 4 and 67 ± 7% of the attached bacteria were released from the nanopatterned PNIPAAM surfaces and the nanopatterned PNIPAAm/QAS composite surfaces, respectively. Neither the bactericidal activity nor the releasing capability was significantly affected after several repeated switching cycles. Similar results were obtained in tests performed on the Gram-positive bacterium, *S*. *epidermidis* [142].

The third strategy to achieve reversible bacteria-killing and detachment switch relies on the counterion-assisted modulation of the interfacial properties, including wettability and zeta-potential of a polycation brush. This concept was conceived by Huang et al. [145] who prepared cationic poly((trimethylamino)ethyl methacrylate chloride) (PTMAEMA) brushes via SIATRP and proved their bactericidal activity against *E*. *coli*, *S*. *epidermidis*, and *Stenotrophomonas maltophilia*. Replacing the chloride counterion by the larger and higher valence hexametaphosphate anion, the binding to the cationic PTMAEMA was reinforced, resulting in “locking” of the counterions and release of the killed bacteria. The initial contact killing status of the brush could be regenerated by simply placing it in a sodium chloride solution for 2 h. Most important, by prolonging the usability of some contaminated items like surgical scalpels, the authors demonstrated the great potential their nanoactuator has for real-world implementation.

## 8. Conclusions and Future Trends

Unlike passive antibiofilm strategies discussed in Part I of the present review, active antibiofilm coatings do not impede bacterial adhesion but rather are designed to prevent biofilm development by killing bacteria or inhibiting their growth. To this purpose, an antimicrobial agent is either covalently tethered to the surface of the coating or physically entrapped within the bulk of the coatings being subsequently eluted from the coated device into the surrounding tissue. The covalent immobilization has the advantage of avoiding the depletion of the biocidal material, but on the other hand, the bactericidal action is restricted to the surface. Another major problem of contact-killing surfaces is the fact that usually, they are thought to be self-deactivating since the corpses of killed bacteria from a surface layer to which new incoming active microbes can adhere and subsequently proliferate on it. The non-covalent immobilization strategy extends the action area of the antimicrobial agent, but at the same time, the effect is limited in time. Moreover, leaching coatings may promote the development of bacterial resistance.

Therefore, the next logical step was to combine the best elements of both passive and active strategies into one single platform. In this combined approach, the two strategies can be applied either simultaneously or sequentially. The appropriate mode of application of the two strategies must take into account their characteristics. For instance, if the contact killing and the antiadhesive strategies are to be combined, it is futile to apply them simultaneously because the active and passive elements dilute each other, and the bactericidal capability will be diminished compared to a pure contact killing surface [128]. Hence, it is much more profitable to apply them sequentially, that is to kill bacteria first and then to release the attached dead bacteria and to prevent further bacterial colonization as described in Section 7 (Applying Active and Passive Strategies One at a Time and Reversibly Switching between the Two Approaches). Through appropriate smart design, the converting process from bactericidal to non-fouling surface can be made reversible, allowing several killing/regeneration cycles to be performed, thereby greatly increasing the antimicrobial efficacy of these surfaces.

Another milestone in the development of antibiofilm surfaces was achieved by designing smart coatings that provide triggered release of the antimicrobial payload in response to some changes in their microenvironment. Internal triggers like pH and temperature, as well as external triggers such as laser light, ultrasounds, and so on, can be used. The problem with this type of triggers is that in some situations, they can activate the release of the antibacterial agent even in the absence of any pathogens. Hence, the antimicrobial compound is uselessly lost and may cause unwanted side effects being also detrimental to beneficial bacteria.

Consequently, a current trend that will continue in the future is the development of smart antibiofilm surface coatings that become activated only in the presence of pathogens. The concept of self-defensive surfaces based on polyelectrolyte multilayers (PEMs) was first demonstrated by Sukhishvili and co-workers [146]. Using the LbL technique, they prepared ultrathin hydrogel films that release antimicrobial agents in response to bacteria-triggered local acidification of the medium. Cado et al. [147,148] prepared PEM films based on the biocompatible and biodegradable polysaccharides HA as polyanion and CHI as polycation, respectively. Using the carbodiimide chemistry and the thiol-maleimide coupling reaction, they grafted bovine cateslytin (CTL), an endogenous antimicrobial peptide, on HA. To this end, cateslytin was previously modified by adding a *C*-terminal cysteine residue (C). The antimicrobial peptide was released from the HA-CTL-C/CHI PEM films due to pathogen-induced hydrolytic biodegradation of HA by hyaluronidase enzymes produced by the Gram-positive *S*. *aureus* bacteria and *Candida albicans* yeasts pathogens. Recently, Yan et al. [149] prepared non-release bacteria-responsive antibacterial surfaces. Their approach relies on a polymer brush with an ingeniously designed hierarchical architecture comprising an inner layer of a covalently immobilized antimicrobial peptide and an outer layer of the pH-responsive poly(methacrylic acid) (PMAA) polymer. In response to pH changes, the outer PMAA layer can be reversible, switched between a deprotonated, hydrated state, which renders the hierarchical surface bacteria resistant and a protonated collapsed state in which the inner antimicrobial peptide layer becomes exposed. Hence, in response to local acidification produced by bacteria, the hierarchical surface exposes the inside bactericidal layer to on-demand kill bacteria.

**Figure 9:**
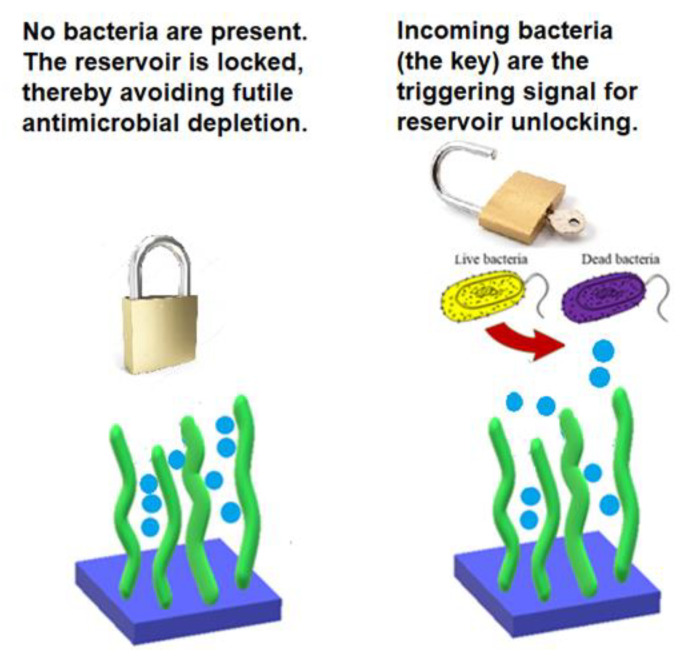
The concept of smart bacteria-activated polymer brush nanocoatings.

We can conclude that future antimicrobial surfaces will consist of smart multifunctional bacteria-activated coatings. The conceptual idea behind this approach is suggestively illustrated in Figure 9.

Despite the important steps taken by the researchers activating worldwide in the field of antibiofilm coatings and surfaces, the domain is still in its infancy, and many discoveries lie ahead. A lot of work remains to be done in the future to fill the existing gap between laboratory testing and real-world applications of anti-infective surfaces.

## Figures and Tables

**Table 1:** Active antibiofilm strategies and the corresponding nanocoatings.

Type of Antimicrobial Agent	Chemical Structure	Mode of Action	Type of Nanocaoting	Type of Clinically Relevant Pathogenic Biofilms/Testing Conditions	Antibiofilm Devices/Biomedical Relevant Outcomes	References
**Polycationic biocides**	6-deoxy-6-(ω-aminoethyl) amino cellulose (AC)	Bacteria-triggered release	Polyelectrolyte multi-layered films of hyaluronic acid (HA)/AC nanospheres constructed by LbL assembly on silicone support	*P*. *aeruginosa*	Urinary catheters	[2]
	Quaternary ammonium poly(ethyleneimine) (QPEI)	Contact-killing	Nanoparticles	*E*. *faecalis*, *S*. *mutans*, *S*. *aureus*, *P*. *aeruginosa*, *S*. *epidermidis*, *C*. *albicans*/both in vitro and in vivo	Surgical dressing materials for wound healing after maxillectomy	[5]
**Antimicrobial peptides (AMPs)**	OP-145	Drug-eluting	Incorporated in a polymer-lipid (PLEX) encapsulation matrix coating on solid medical-grade TAN	*S*. *aureus*/in vivo animal models (mice and rabbits)	Prevention of *S*. *aureus* implants-associated osteomyelitis	[9]
	14-helical β peptide	Drug-eluting	Embedded in polyelectrolyte multi-layer coating constructed by LbL self-assembly of alternating layers of HA/chitosan (CHI) or poly-*L*-glutamate (PGA)/poly-*L*-lysine (PLL)	*C*. *albicans*/in vitro in synthetic urine (SU) media	Urinary catheters	[11]
	RK1 (RWKRWWRRKK) RK2 (RKKRWWRRKK)	Contact-killing	Covalently tethering of RK1 to an allyl glycidyl ether (AGE) polymer brush on silicone (polydimethylsiloxane; PDMS) support	*E*. *coli*, *S*. *aureus* and *C*. *albicans* in both phosphate buffer saline (PBS) and urine media	Prevention of catheter-associated urinary tract infections (CAUTIs)	[13]
	Lassioglosin III	Contact-killing	Covalent linking of the AMP to allyl AGE) polymer brush on PDMS support	*E*. *coli*, *E*. *faecalis* biofilms in SU environment	Silicone Foley catheters	[14]
	CWR11	Contact-killing	Covalent immobilization of synthetic CWR11 antimicrobial peptide on PDMS support via an intermediate crosslinking mussel-inspired polydopamine (PDA) film	*E*. *coli*, *S*. *aureus*, and green fluorescent protein (GFP)-*P*. *aeruginosa* in air, PBS, SU, and urine media	Foley urinary catheters	[15,16,17]
**Antimicrobial enzymes**	Cellobiose dehydrogenase (CDH)	Generation of hydrogen peroxide (H_2_O_2_)	Covalent immobilized CDH on amine modified PDMS support via a homodifunctional glutaraldehyde (GA) crosslinker	*S*. *aureus* under dynamic conditions in an artificial bladder system model	Urinary catheters	[22]
	Deoxyribonuclease I (DNase I) and Ribonuclease A (RNaseA)	Matrix eDNA degradation, Inhibition of bacteria adhesion due to RNase A hydrophilicity	Surface covalent immobilization of nucleases on poly(2-carboxyethyl acrylate) (PCA) polymer brush grafted from a tri-block thermoplastic elastomer poly(styrene-b-isobutylene-b-styrene) (SIBS) support by surface-initiated polymerization (SIP)	*S. aureus*, *E. coli* biofilms	Strong inhibition of *S. aureus* biofilms by DNase I and of *E*. *coli* biofilms by RNase A; prevent adhesion and formation of a conditioning film and further bacterial colonization	[23]
	Deoxyribonuclease I (DNase I)	Matrix eDNA degradation	Covalent immobilization of DNase I on polymethylmethacrylate (PMMA) support via a PDA film	*P*. *aeruginosa* PAO1, *S*. *aureus* ATCC 12,600	Strong inhibition of biofilm growth	[24]
**Antibiotics**	Amikacin, Vancomycin	Drug-eluting	Encapsulated in a phosphatidylcholine lipid matrix	Polymicrobial mixture of *S*. *aureus* and *P*. *aeruginosa*/in vivo in a mouse model	Prevention of implant-associated infections	[25]
	Doxycycline	Drug-eluting	Polymer-lipid encapsulation matrix (PLEX)	Methicillin-resistant *S*. *aureus* (MRSA) /in vivo in a rabbit intramedullary nail model	Prevent implant-associated osteomyelitis	[26]
	Minocycline hydrochloride (MH)	Drug-eluting	Hydrophilic multilayer coatings with embedded MH prepared by the LbL self-assembly of the anionic polyelectrolite dextransulfate (DS)/cationic gelatin type A (GA) polyelectrolyte in the presence of Ca^2+^ ions	*E*. *coli* ATCC 25922, *E*. *coli* 0157:H7, *A*. *baumannii* ATCC 19606, *S*. *aureus* ATCC 25923, MRSA USA300, *S*. *epidermidis* ATCC 12228, and a clinical isolate *A*. *baumannii*	Complete elimination of multi-drug resistant *A*. *baumannii* biofilm A pH-triggered smart drug release system for targeted delivery of the broad-spectrum antibiotic and anti-inflammatory drug MH	[28]
	Ciprofloxacin hydrochloride (CH)	Drug-eluting	Encapsulated in polyelectrolyte multilayer microcapsules with pH-tunable permeability fabricated by LbL deposition of poly(allylamine hydrochloride) (PAH) and poly(methacrylic acid (PMA) on poly(styrene sulfonate) (PSS)-doped calcium carbonate particles as sacrificial templates	*E*. *coli*	Sustained release of CH resulting in biofilm inhibition	[35]
	Gentamicin	Drug-eluting	Embedded in hydroxyapatite (HAP) coating on titanium alloy (Ti-6Al-4V) covered with a protective, biodegradable polyester copolymer poly(lactic-co-glycolic acid) (PLGA) overlayer	*S*. *epidermidis* 7388 and *S*. *epidermidis* 7391 strains/in vivo in a rabbit model	Prophylaxis of bone implant-associated infections	[36]
	Ceftaroline fosamil or Zinforo (ZiN)	Drug-eluting	Incorporated in mesoporous silica nanocoatings deposited by matrix assisted pulsed laser evaporation (MAPLE) on various substrates (double side polish silicon, glass, and commercial pure Ti grade 2 disks)	*E*. *coli* ATCC 25922/in vitro on cultured human cells, and in vivo using a mouse model	Strong antibiofilm activity against *E*. *coli* during the early stage of biofilm formation	[37]
	Cefotaxime (sodium salt; CS)	Non-leaching antibiotic covalently tethered surface	Covalent immobilized CS on a Ti substrate through an intermediary adhesive anchoring PDA primer	*E*. *coli* and *S*. *mutans* biofilms	Applications in dental and bone graft substitutes	[38]
***N*-halamines**	2,2,6,6-tetramethyl-4-piperidinol-based *N*-chloramine	Combined contact-killing and release-killing mechanisms	Covalently immobilized *N*-halamine on the surface of hybrid silica-polystyrene (PS) nanoparticles with well-defined core-shell morphology	*E*. *coli*	Complete inhibition of bacterial colonies growth	[40]
**Chlorhexidine (CHX)**	Chlorhexidine digluconate	Drug-release	Immobilization of CHX on stainless steel using crosslinking polydopamine film	*E*. *coli* and *S*. *aureus*	High antibacterial efficacy against both bacteria, even at a low concentration of CHX	[45]
**Usnic acid (UA)**	2,6-Diacetyl-7,9-dihydroxy-8,9b-dimethyldibenzo[b,d]furan-1,3(2H,9bH)-dione	Drug-release	UA physically adsorbed on magnetically targetable superparamagnetic Fe_3_O_4_ nanoparticles stabilized by an oleic acid shell	*S*. *aureus* biofilm	Increased antibiofilm activity	[47,48]
		Drug-release	Incorporated in MAPLE deposited thin film coatings of polylactic acid (PLA)-polyvinyl alcohol (PVA) microspheres loaded with UA	*S*. *aureus* biofilm	Inhibitory activity on early and mature *S*. *aureus* biofilms	[49]
		Drug-release	MAPLE deposited thin films consisting of inclusion complexes of UA with γ-cyclodextrin (γ-CD) on double-side polished silicon and glass slides supports	*S*. *aureus* biofilm	Hinder bacterial attachment and biofilm formation for at least 72 h	[50]
**Quorum sensing inhibitors (QSIs)**	(Z-)-4-bromo-5-(bromomethylene)-2(5H)-furanone	Drug-eluting	Incorporation of the QSI halogenated furanone compound into a Nafion polymer matrix coating on glass support	Mixed microbial culture and pure strains of *P*. *aeruginosa*, *E*. *coli*, and *B*. *subtilis*	Important biofilm reduction	[51]
	Dihydropyrrolones (DHPs)	Inhibition of OS probably through a membrane mediated mechanism	Covalent attachment of potent QS inhibitors dihydropyrrolones to a glass substrate via a click-chemistry constructed 1,2,3-triazole ring linker	*P*. *aeruginosa*, *S*. *aureus* /in vitro	72 percent repression of *lasB* gene expression in *P*. *aeruginosa* Reduction of bacterial adhesion up to 97% for both *P*. *aeruginosa* and *S*. *aureus*	[52]
**Modulators of c-di-GMP-dependent signal transduction pathway**	*N*-diazeniumdiolates (NONOates)	Releasing of nitric oxide (NO) biofilm dispersal signal	*N*-succinyl chitosan sodium (CPCS) cross-linked to branched poly(ethyleneimine) (PEI) NONOate NO donor nanogel conjugates	*S*. *aureus* and *E*. *coli*	excellent inhibition of both biofilms sustained NO release	[70]
		Inhibition of bacterial proliferation	Incorporation of the lipophilic dibutylhexyldiamine diazeniumdiolate (DBHD/N_2_O_2_) into the hydrophobic, biodegradable, and biocompatible polymer PLGA coated with a protective top coating of silicone rubber (SR)	*S*. *aureus* and *E*. *coli*	biofilm biomass reduction by about 98% for *S*. *aureus* and up to 99.9% for *E*. *coli* tunable NO release rate	[71]
		Killing bacteria	Glass substrate coated with a PDA film modified through pegylation and functionalized with NONOates	*P*. *aeruginosa* (wild-type PAO1 and multidrug-resistant PA37) and *S*. *aureus* (ATCC 29213)	Biofilm development inhibition by 96% at 24 h, and by 70% at 36 h High killing efficiency	[72]
**Silver**	Zero-valent silver and silver ions	Drug-releasing	Alginate stabilized silver nanoparticles (SA-AgNPs)	*L*. *monocytogenes*, *V*. *parahaemolyticus*	Biofilm inhibition of 98% for *L*. *monocytogenes* and of 84% for *V*. *parahaemolyticus*	[112,113]
			*Heliotropium crispum* (HC) plant extract capped silver nanoparticles (HC-AgNPs)	Clinical isolates of MRSA, *P*. *aeruginosa*, and *A*. *baumannii*	Biofilm disruption, changes in the morphology of bacteria and destruction of the extracellular polymeric substances (EPS)	[114]
			Incorporation of silver ions doped TiO_2_ into a polymeric epoxy matrix	*E*. *coli* and *S*. *aureus*	Complete biofilm inhibition for both pathogens	[115]
